# Bacteriophages mobilize bacterial defense systems via lateral transduction

**DOI:** 10.1126/sciadv.adx5749

**Published:** 2026-01-23

**Authors:** Xu Kuang, Jamie Gorzynski, Marie Touchon, Andrey Shkoporov, Eduardo P. C. Rocha, J. Ross Fitzgerald, John Chen, Jakob T. Rostøl, José R. Penadés

**Affiliations:** ^1^ Department of Infectious Disease, Imperial College London, London SW7 2AZ, UK.; ^2^ Centre for Bacterial Resistance Biology, Imperial College London, London SW7 2AZ, UK.; ^3^ The Roslin Institute, University of Edinburgh, Edinburgh, UK.; ^4^ CNRS, UMR3525, Microbial Evolutionary Genomics, Institut Pasteur, Université de Paris Cité, 75015 Paris, France.; ^5^ School of Microbiology, University College Cork, Cork, Ireland.; ^6^ Department of Microbiology and Immunology, Infectious Diseases Translational Research Programme, Yong Loo Lin School of Medicine, National University of Singapore, Singapore 117597, Singapore.; ^7^ Universidad CEU Cardenal Herrera, Valencia, Spain.

## Abstract

To counter challenges from bacteriophages (phages), bacteria use defense mechanisms that can reside on mobile genetic elements or within chromosomes. These immune systems are easily gained and lost, allowing adaptation to threats. However, the mechanism of mobilization of chromosomally encoded defense genes remains poorly understood. Here, we show that phage- and phage-inducible chromosomal island (PICI)–mediated lateral transduction (LT), a highly efficient horizontal gene transfer mechanism, facilitates the transfer of these defense genes between bacteria. Using several bacterial models, we demonstrate that defense systems are often positioned near phage or PICI attachment sites, allowing them to exploit LT for their mobility. In addition, LT diversifies defense genes carried by prophages and PICIs, driving immune system evolution and turnover. These processes provide phage resistance to new bacterial hosts and profoundly affect population genomics. Our findings reveal LT as a crucial mechanism shaping bacterial evolution and influencing the trajectory of pathogenic clones in nature.

## Teaser

Bacteriophages and PICIs spread bacterial defenses via lateral transduction, shaping microbial immunity and pathogen evolution.

## INTRODUCTION

Bacteriophages (phages) are viruses that infect and replicate within bacteria, representing a major threat to bacterial populations. In response, bacteria harbor a large set of defense genes, collectively referred to as antiphage systems, which work through diverse mechanisms to prevent the invader from replicating and killing the host (*1*–*7*). For example, two widespread mechanisms are restriction-modification (R-M) systems, which cleave foreign, unmethylated DNA (*8*), and CRISPR-Cas systems, an adaptive system that targets invader DNA in a sequence-specific manner (*9*, *10*). Antiphage systems are important players in the ongoing coevolutionary arms race between bacteria and phages (*11*), which drives ecological and evolutionary processes in microbial communities, including pathogens (*12*–*14*).

To counter diverse phages, many of which can evade defenses, bacteria typically encode multiple immune systems that can provide additive or synergistic protection (*15*–*18*). Genomic analysis has revealed that antiphage systems tend to cluster together into “defense islands” (*4*, *19*, *20*), possibly to allow joint transfer. However, because of autoimmunity and metabolic burdens, there is a limit to the number of systems harbored by one bacterium. Instead, the pan-immune system (*21*), the collection of available defense genes available to a population, allows the bacterial population to quickly adapt to new threats. As such, closely related strains often encode different defense mechanisms, and studying evolutionary dynamics reveals that defense systems are rapidly gained and lost in populations (*22*, *23*). For many immune genes, their mobility is explained by their presence within mobile genetic elements (MGEs) such as temperate phages, plasmids, and phage satellites (*24*–*26*). However, many antiphage genes and defense islands have also been identified in the chromosome outside of integrated MGEs. For these, the mechanism(s) of mobility remains poorly understood. Yet, the apparent modularity of these defense islands within their chromosomal contexts suggests that they were acquired through homologous recombination. In this case, the mechanism of external DNA delivery remains unclear.

Recently, a powerful mechanism of horizontal gene transfer (HGT), lateral transduction (LT), was found (*27*, *28*). LT occurs when an induced prophage starts DNA packaging while the prophage is still integrated in the chromosome, leading to multiple infecting phage–like particles being packaged by chromosomal DNA downstream of the phage attachment site (*attB*). As a result, large regions of bacterial DNA are released in transducing particles and can be transferred to susceptible recipients, where the DNA can be integrated through homologous recombination. LT was initially found in *Staphylococcus aureus* (*27*, *28*) but has since been recognized as a hallmark of temperate phages in many bacterial species (*27*–*31*). More recently, members of the phage satellite family phage-inducible chromosomal islands (PICIs) have also been shown to mediate LT (*32*). PICIs package DNA in a similar way to phages and can thus mobilize DNA downstream of their specific *attB* sites. PICIs can also engage in lateral cotransduction (LcT), a process where recombination occurs between PICI genomes replicating in parallel (*32*). Using the PICI members present in *S. aureus* [commonly referred to as *S. aureus* pathogenicity islands (SaPIs)], it was observed that following SaPI induction, the SaPI replicates while still integrated in the bacterial chromosome (in situ replication), generating multiple copies of its genome. While some of these copies excise from the chromosome through the activity of the SaPI-encoded excisionase (Xis) and integrase (Int) proteins, which recognize specific attachment sites (*attL* and *attR*) at the ends of the SaPI genomes, in some instances, the Int/Xis proteins catalyze recombination using the *attR* site from one SaPI genome and the *attL* site from another. This process results in tandem SaPI genomes within the bacterial chromosome. During in situ packaging, when the *pac* site of the first SaPI is targeted, the DNA packaged in the first capsid contains both an intact PICI genome and downstream chromosomal DNA within the same infective particle (*32*).

Here, given that LT and LcT can transfer large chromosomal regions, we hypothesized that they might be responsible for mobilizing chromosomally encoded antiphage systems and defense islands between bacteria. We suspected that many immune genes are encoded downstream of phage/PICI *attB* sites, allowing them to hijack LT/LcT for their own mobility, a process that would explain their observed variability across genomes. Moreover, with the large packaging capacity of phage capsids, we hypothesized that even large defense islands might be moved to new hosts.

Our results confirm these hypotheses, and we show that chromosomally encoded defense islands are packaged via LT in natural environments at high efficiencies, a process that promotes their transfer to naïve hosts. This process not only offers protection to recipient cells from phage predation but also determines their evolutionary trajectory. In addition, we show that LT facilitates the exchange of phage- and PICI-encoded genes to imbue these elements with new antiphage properties, and we observe that antiphage and virulence genes are often located in prophage and PICI regions that can be diversified through LT. Overall, we demonstrate that LT and LcT play a key role in the dissemination of bacterial defense systems throughout bacterial populations and are central processes not only in the host-phage arms race but also in the evolution and emergence of novel bacterial pathogens.

## RESULTS

### *S. aureus* defense islands are located next to phage and PICI *attB* sites

**Fig. 1.**
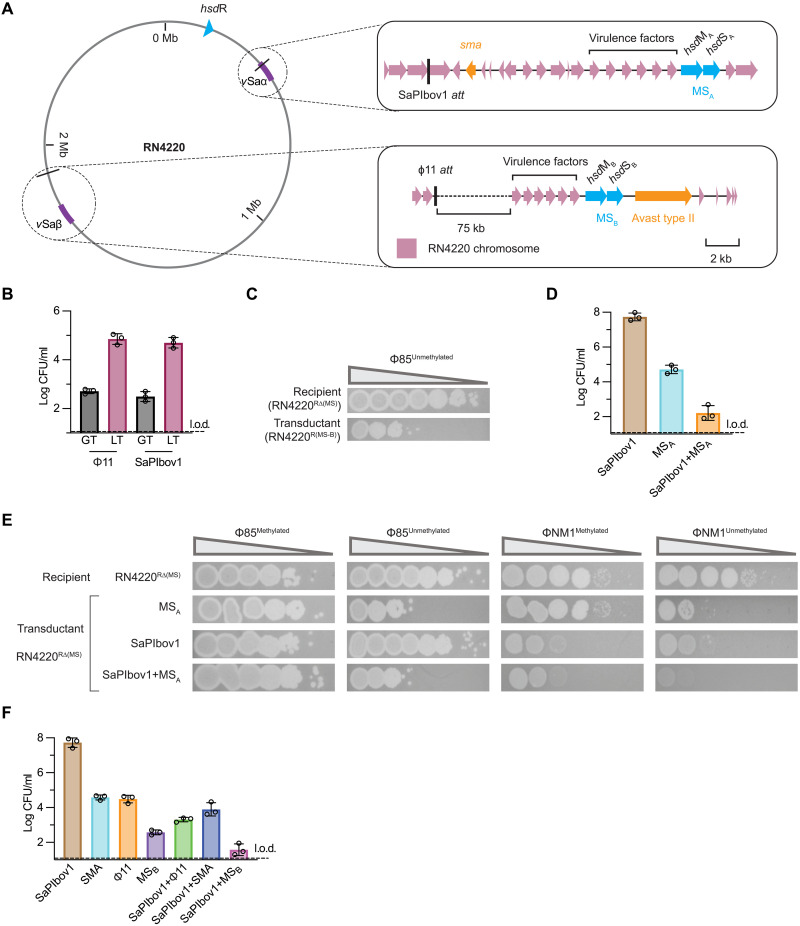
Mobilization of *S. aureus* defense islands via phage- and PICI-mediated LT. (**A**) Schematic of the *S. aureus* RN4220 chromosome showing R-M genes MS_A_ and MS_B_ in pathogenicity islands *v*Saα and *v*Saβ, respectively, downstream of the SaPIbov1 and Φ11 *attB* sites. Virulence factors and the additional defense genes *sma* and *avs2* (Avast type II) are also depicted. (**B**) The efficiency of Φ11-mediated LT and GT transferring the Cd resistance marker near MS_B_ from donor RN4220 to recipient RN4220^RΔ(MS)^ is shown on the left. The right displays the efficiency of SaPIbov1-mediated LT and GT for transferring the Cd resistance marker near MS_A_. Results are log CFU per milliliter with the limit of detection (l.o.d.) indicated. Bars represent the means of three biological replicates ± SD. (**C**) Spot assay demonstrating phage resistance in a transductant from LT in (B). The RN4220^RΔ(MS)^ transductant with MS_B_ blocks infection by unmethylated Φ85, confirming restoration of R-M protection. Tenfold phage dilutions were spotted with decreasing titer indicated by the triangle. The image is representative of three biological replicates. (**D**) Efficiency of SaPIbov1 transfer (brown), SaPIbov1-mediated LT for transferring MS_A_ (light blue), and both SaPIbov1 and MS_A_ transfer (orange) from RN4220 to RN4220^RΔ(MS)^. Results are log CFU per milliliter with l.o.d. indicated, showing recipients with the tetracycline resistance marker in SaPIbov1, the Cd resistance marker near MS_A_, or both. (**E**) Functional analysis of LT and LcT transductants against unmethylated or methylated Φ85 or ΦNM1. Transductants with MS_A_ or MS_A_ + SaPIbov1 show distinct phage resistance patterns, including additive protection against unmethylated ΦNM1. Tenfold phage dilutions were spotted, with decreasing titer indicated by the triangle. (**F**) Quantification of comobilization of multiple defense systems from a donor harboring phage Φ11 (erythromycin marker), SaPIbov1 (tetracycline marker), and chromosomal markers near *sma* (*v*Saα) and MS_B_ (*v*Saβ). Enumerated individual and combinatorial transductants are shown. See fig. S3D for spot assays using these transductants.

We started this study using *S. aureus* for several reasons. First, it is an important human and animal pathogen where HGT plays a key role (*33*–*35*). Second, PICIs (SaPIs in *S. aureus*), LT, and LcT were all found in this bacterium (*27*, *32*, *36*). Last, most *S. aureus* strains encode two different type I R-M systems, which act as important barriers of genetic transfer (*37*). The classical *S. aureus* lab strain RN4220 is defective in this activity (*38*), helping to explain this strain’s ability to take up plasmid DNA and be infected by many phages. Type I R-M systems, *Sau*I, consist of three *hsd* (host specificity for DNA) genes, *hsdR*, *hsdM*, and *hsdS*, encoding proteins responsible for restriction, modification, and target sequence specificity, respectively. The holoenzyme M_2_S_1_ is responsible for methylating self-DNA (to prevent autoimmunity), while R_2_M_2_S_1_ translocates and cleaves unmethylated (foreign) DNA (*39*). Most *S. aureus* strains have two genomic locations involved in R-M target specificity, each one encoding a different *hsdS* allele (Fig. 1A). Therefore, this bacterium can recognize and either methylate or cleave two different DNA sequences.

The importance of type I R-M systems in *S. aureus* is highlighted by their role in establishing its genetic structure. *S. aureus* has multiple clonal complexes (CCs), which are crucial in understanding and explaining the epidemiology, evolution, and pathogenicity of this species (*40*). A CC refers to a group of bacterial strains that are genetically related and descend from a common ancestor and often infect specific animal hosts. While most *S. aureus* strains carry the same *hsdR* gene, different alleles of the *hsdM* and *hsdS* genes are found in different CCs (*41*). Each CC is thought to encode a particular combination of *hsdM* and *hsdS* genes. Given that DNA methylation patterns from one *hsdM*/*hsdS* background are likely to be targeted by the R-M system of a different CC, this may explain why MGEs more readily transfer within CCs than between CCs.

In *S. aureus*, the *hsdM*/*hsdS* genes cluster together and are present in pathogenicity islands *v*Saα and *v*Saβ (fig. S1), islands that carry multiple exotoxins and virulence factors. By contrast, *hsdR* is located elsewhere in the chromosome (Fig. 1A). Given that antiphage systems often cluster together, we first analyzed whether there were additional immune systems in the vicinity of the *hsd* genes in RN4220. In *v*Saα, near *hsdM*/*hsdS* (referred to as MS_A_), we detected SMA (single-protein MazF-like antiphage system), and in *v*Saβ, near *hsdM*/*hsdS* (referred to as MS_B_), we observed the presence of Avast [antiviral ATPase (adenosine triphosphatase)/NTPase (nucleoside triphosphatase) of the STAND superfamily] type II. *v*Saα and *v*Saβ therefore, in addition to being reservoirs for toxin genes as pathogenicity islands, also provide antiphage immunity by being defense islands.

### Defense islands can be mobilized by phage-mediated LT

Given that we previously showed that *v*Saβ and *v*Saα are localized next to the prophage ϕ11 and SaPIbov1 *attB* sites (Fig. 1A) (*27*, *32*), we hypothesized that the defense island carrying the *hsdM*/*hsdS* genes could be mobilized by LT. We first investigated the movement of *hsdM*/*hsdS* located in *v*Saβ, referred to as MS_B_, downstream of the ϕ11 *attB* site. To this end, we inserted a cadmium (Cd) marker near MS_B_ in strain RN4220 (donor), induced the ϕ11 prophage through the addition of the DNA damaging agent mitomycin C (MC), and quantified the transfer of the marker with the adjacent MS_B_ genes into an RN4220 strain with MS_A_ and MS_B_ deleted [RN4220^RΔ(MS)^]. As a control for generalized transduction (GT), we used a separate marker located elsewhere in the chromosome (Materials and Methods and fig. S2). Our experiments showed that the marker downstream of ϕ11 could be transferred efficiently into RN4220 (Fig. 1B), with rates higher than that of GT. We confirmed that MS_B_ movement co-occurred with the marker by testing transductants for the MS_B_ presence by polymerase chain reaction (PCR) (fig. S3A). To test whether the transfer of MS_B_ into RN4220^RΔ(MS)^ provided immunity, we challenged the resulting cells (transductants) with unmethylated ϕ85 (ϕ85^Unmethylated^). Compared to the untreated RN4220^RΔ(MS)^ recipient, the transductant was able to strongly block phage infection (Fig. 1C), confirming that the R-M system is functionally restored by the LT transfer of MS_B_.

### Mobilization of defense islands via PICI-mediated LT and LcT

Having confirmed that phage-mediated LT could mobilize functional antiphage genes, we next sought to investigate whether PICI-mediated LT/LcT could play an analogous role in defense gene transfer. In strain RN4220, given that the SaPIbov1 *attB* site is upstream of *v*Saα, which includes *hsdM*/*S* (MS_A_) and SMA antiphage genes, we placed a Cd marker upstream of MS_A_. The donor strain also harbored SaPIbov1 with a tetracycline resistance marker (*tst*::*tetM*) and helper phage 80α with the small terminase (*terS*) gene deleted (80α^Δ*terS*^). Upon MC addition, 80α^Δ*terS*^ is induced, which activates the SaPIbov1 life cycle, while the terminase mutant prevents mature 80α particles from forming, thereby preventing phage-mediated transduction. The RN4220^RΔ(MS)^ strain was again used as the recipient, and a GT control with a Cd marker elsewhere in the chromosome was included (Materials and Methods). In support of our hypothesis, enumerating Cd-resistant transductants revealed high rates of LT into the recipient strain (Fig. 1B), confirming that the region downstream of the SaPIbov1 site, including MS_A_ (fig. S3B), can be transferred between strains. To confirm that the transferred MS_A_ was functional, we challenged transductants with ϕ85^Unmethylated^ in a spot assay, which confirmed that MS_A_ is active and provides phage protection together with *hsdR* (fig. S3C).

### LcT provides phage protection through two mechanisms

Given that SaPIbov1 can engage in similar but distinct transfer mechanisms, LT and LcT (*32*), we next quantified the relative contributions of each mechanism to transfer. To distinguish between LT and LcT, we quantified the number of Cd-resistant transductants that were also tetracycline-resistant, indicative of the concomitant transfer of MS_A_ and SaPIbov1 through LcT. This was compared with the number of transductants that were only resistant to Cd, indicative of LT. We also quantified the number of cells only carrying SaPIbov1 *tst*::*tetM*. As previously reported, SaPIbov1 transfer was extremely high, more than two orders of magnitude higher than the transfer of the MS_A_ genes by LT (Fig. 1D). The concomitant transfer of these elements via LcT also occurred in a subset of the population (Fig. 1D).

PICIs can interfere with phage reproduction by hijacking the phage machinery for packaging and transfer after induction (*42*) and by encoding specific antiphage systems whose function is not linked to the PICI cycle (*25*). The fact that LcT mobilizes not only the defense islands but also the PICIs themselves raises the possibility that recipient cells receiving LcT obtain additive protection through distinct mechanisms. To test this, we challenged different transductants with unmethylated and methylated phage ϕNM1 (a helper phage of SaPIbov1) and ϕ85 (a nonhelper phage). We observed distinct protection patterns: In the case of LT, the restored MS_A_ system provided protection against both unmethylated phages, while SaPIbov1 protected against ϕNM1 regardless of methylation state (Fig. 1E). Notably, against ϕNM1^Unmethylated^, defense was additive, being stronger than with R-M or SaPIbov1 protection alone. LcT therefore has the potential to transfer both chromosomally encoded antiphage systems and PICIs that defend from phage predation. From the perspective of the PICI, it benefits from “traveling together” with MS_B_, being protected by these genes in its new host.

### Diversity in immune system transfer enhances phage defense

It has been proposed that within a population, to avoid the cost associated with their carriage, immune systems (pangenome elements) may be distributed across different cells, which can then share them to generate strains capable of responding to various attacks (*21*). The previous data (Fig. 1, D and E) provide a demonstration that this process can occur naturally through parallel transfer mechanisms.

To further investigate this concept, and considering that a typical *S. aureus* strain harbors three prophages and one SaPI, we explored the possibility that multiple defense genes, either chromosomally encoded or carried on MGEs, could be mobilized simultaneously. This would create substantial variability in the recipient cells, enabling them to obtain and carry unique combinations of defense systems that can respond differentially to phage attacks. To test this hypothesis, we engineered a strain carrying phage ϕ11 (encoding *ermR*), SaPIbov1 (*tetR*), and Cm and Cd markers adjacent to the *sma* (*v*Saα) and MS_B_ (*v*Saβ) genes. The strain was MC induced, and the transfer of the different markers was monitored.

Supporting our hypothesis, a mixed population of recipients was generated when transferring an MC-induced lysate of the engineered strain to RN4220^RΔ(MS)^ (Fig. 1F). These cells carried different combinations of the studied defense systems at varying frequencies. Cells simultaneously harboring multiple immune systems were obtained, enabling them to respond differently to attacks by diverse phages (fig. S3D). In summary, our results reveal an elegant combination of natural mechanisms by which bacterial populations orchestrate their defenses, arming themselves with unparalleled adaptability against viral threats.

### Inter-CC mobilization of defense islands via LT and LcT

So far, all the experiments performed to analyze the power of LT and LcT in mobilizing DNA between strains used RN4220 or RN450 derivatives as donors and recipients, belonging to CC8. RN4220 is an RN450 derivative mutant carrying, among others, a mutation in the *hsdR* gene, which makes this strain ideal for accepting exogenous DNA (*38*). It is expected, however, that when donor and recipient cells belong to different CCs, the methylation pattern of the donor DNA will be recognized as foreign by the recipient, potentially limiting or even eliminating the acquisition of foreign DNA. In addition, for the *v*Saα or *v*Saβ islands, not only can the *hsdM/hsdS* genes differ, but there are also multiple allelic variants of the toxin genes present in these islands that vary between CCs. Given that both LT and LcT require homologous recombination in the recipient, differences in DNA sequence between the donor and recipient cells may prevent recombination and impair the transfer of defense systems between CCs.

To determine whether LT or LcT of the *hsdM/hsdS* genes can occur when the donor and recipient are not from the same CC, we used the same donor strain as before—RN4220 derivatives belonging to CC8 with Cd markers next to the MS_A_ or MS_B_ genes, which can be mobilized by PICI (SaPIbov1) or phage (Φ11)–mediated LT, respectively. As recipients, we used eight clinical isolates belonging to different CCs (including CC8) that carry different combinations of MS_A_ and MS_B_ genes (table S1). While some of these CCs were associated with human infections (CC8, CC121, CC1, CC15, and CC5), others belonged to strains infecting nonhuman hosts (ST133, ST130, and CC395), highlighting their diversity. As a control in these experiments, we also measured the transfer of different R-M systems via GT.

**Fig. 2.**
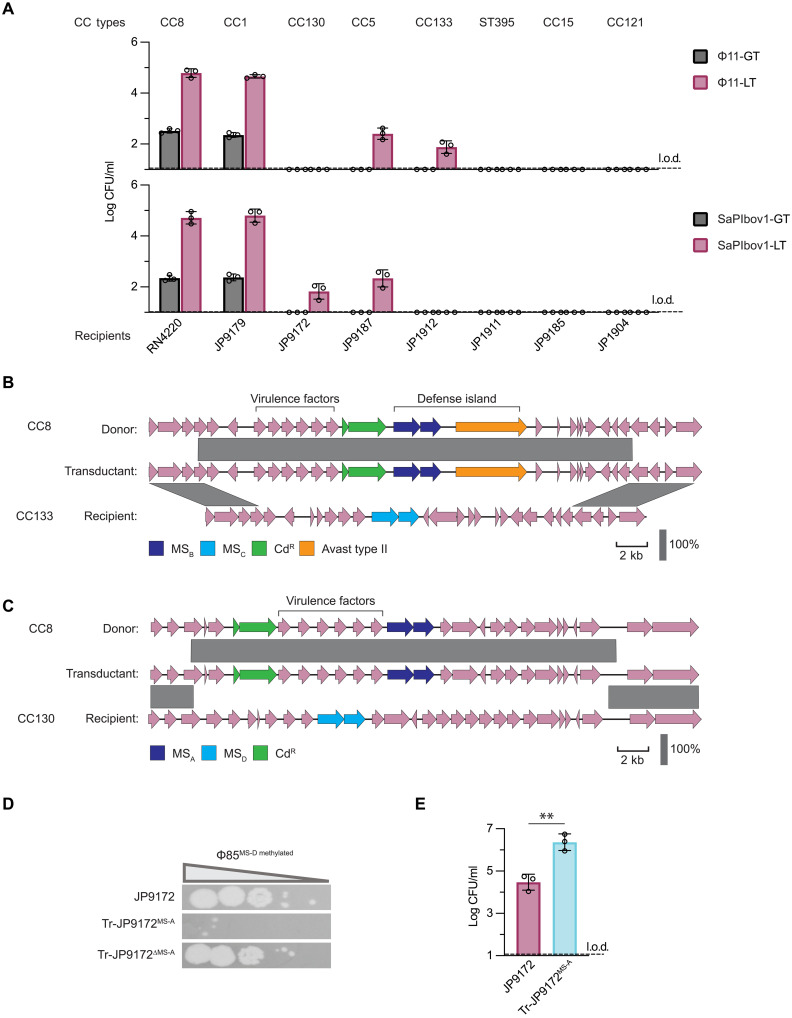
LT can mobilize DNA between different *S. aureus* CCs. (**A**) Transfer of defense islands via LT to recipient strains from different CCs. Lysates of *S. aureus* RN4220 derivatives harboring Φ11 for MS_B_ movement or SaPIbov1 for MS_A_ movement were transduced and transferred into eight clinical isolates belonging to diverse CCs. Cd resistance markers were used to quantify transduction efficiencies. Data are provided as log-transformed CFU per milliliter of LT lysate. Each bar represents the means of three biological replicates ± SD. (**B**) Genomic organization of the locus surrounding MS_B_ transferred from the donor to the recipient via Φ11-mediated LT. A large genomic region from the donor was revealed by WGS to be transferred to the recipient strain JP1912, generating a chimeric transductant. The scale bar represents 2 kb. (**C**) As in (B) but with SaPIbov1-mediated LT transfer of the region encoding MS_A_ into recipient JP9172. (**D**) Using Φ85 passaged on strain JP9172 (Φ85^MS-D methylated^) to infect the recipient JP9172 with the original MS_D_, the transductant JP9172 from (C) (Tr-JP9172^MS-A^), or Tr-JP9172^MS-A^ with MS_A_ deleted (Tr-JP9172^ΔMS-A^). Tenfold phage dilutions were spotted, with decreasing phage titer indicated by the triangle. The image is representative of three biological replicates. (**E**) Determining the transfer efficiency of SaPIbov1 into either JP9172 (with the original MS_D_) or transductant Tr-JP9172^MS-A^ (with MS_A_). Data are provided as log-transformed CFU per milliliter of LT lysate. Adjusted **P = 0.0039 (unpaired *t* test). Each bar represents the means of three biological replicates ± SD.

Of the eight strains used as recipients, we demonstrated inter-CC phage-mediated transfer of MS_B_ in four of the isolates, with transfer to the strain belonging to CC1 occurring at the same high level as observed when the CC8 strain was used as the recipient (Fig. 2A). Transfer via GT occurred at a low frequency and only in the two recipient strains that showed high transfer via LT, indicating that LT is not only more efficient in terms of transfer frequency but also in the range of recipient strains to which the R-M systems can be mobilized.

Given that the donor and recipient strains carry different *hsdM/hsdS* alleles (table S1), we confirmed the transfer of MS_B_ alleles from RN4220 (CC8) to strains belonging to CC133, CC1, and CC5, which carry different alleles (here named MS_C_, MS_JP9179_, and MS_JP9187_, respectively), by performing whole-genome sequencing (WGS) on three transductants. This showed that the recipients had swapped their *hsdM/hsdS* genes, now carrying the MS_B_ variant from the CC8 donor (Fig. 2B and fig. S4, A and B). The Avast type II system from the donor defense island was also exchanged, as were the toxin genes flanking the defense island, demonstrating how a phenotypically significant DNA region of more than 10 kb can be transferred between CCs.

We obtained similar results when the transfer of MS_A_ via SaPIbov1-mediated LT was evaluated. Like before, transfer of MS_A_ to strains belonging to CC1 occurred at a high frequency via LT and at a much lower frequency via GT, and LT could also move DNA to CC5. However, unlike phage-mediated LT, which could transfer to CC133, PICI-mediated LT could instead serve as a donor to CC130 (Fig. 2A). Sequencing one of the transductants obtained when the strain belonging to CC130 was used as a recipient confirmed the formation of a chimeric strain carrying not only the R-M systems from the donor (the MS_A_ replaced the preexisting MS_D_) but also the toxin genes adjacent to these systems (Fig. 2C). The phage resistance profile of the transductant was modified as a consequence of acquiring a new R-M system. While the original strain carrying the MS_D_ system was susceptible to phage 80α propagated on this strain (carrying methylation patterns from MS_D_), the chimeric strain was almost completely resistant to that phage (Fig. 2D). This resistance depended on the newly acquired MS_A_ genes, as deletion of these genes restored phage infectivity. Overall, these results confirm not only the power of LT in mobilizing immune systems but also its role in creating new variants with the ability to differentially block phage attack.

Last, we wondered whether there could be a direct benefit to the PICI by performing LT of *hsdM*/*hsdS* genes. When SaPIbov1 is produced from RN4220, its genome is methylated by MS_A_, which protects it from restriction by cells containing an R-M system with MS_A_. Host cells with different methylation patterns from other CCs, like strain JP9172 (with MS_D_), would restrict incoming SaPIbov1. To avoid this, we first made SaPIbov1 transfer MS_A_ to the recipient cell, generating a transductant without MS_D_. We then observed that the subsequent transfer of SaPIbov1 into this chimeric JP9172(MS_A_) strain was significantly more efficient (Fig. 2E). In other words, SaPIbov1 can enhance its own dissemination by first priming its future recipient to not block transfer via R-M.

### Mobilization of defense islands via LT and LcT shapes the *S. aureus* genomic structure

**Fig. 3.**
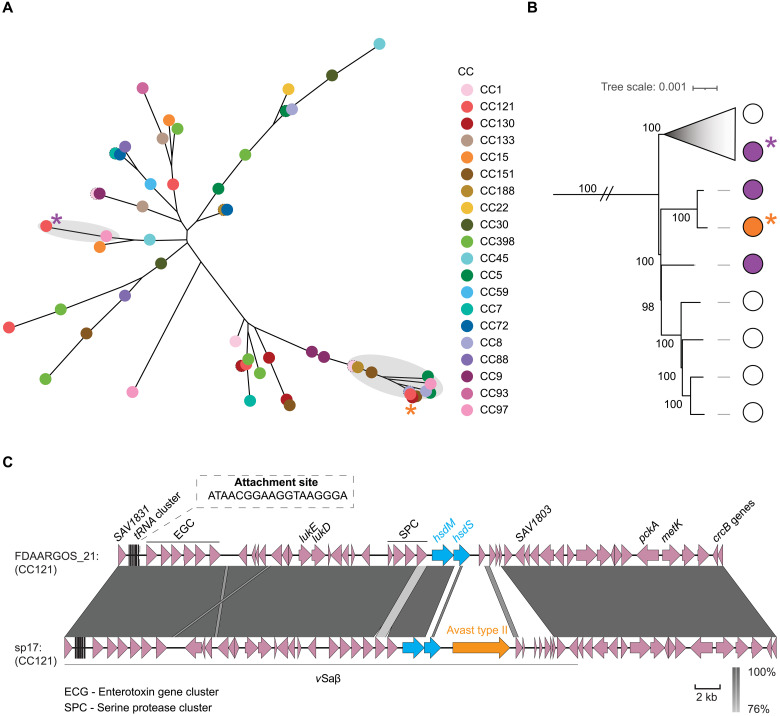
Horizontal transfer of *SauI* variants into chromosomal regions with a high LT frequency. (**A**) Unrooted phylogenetic tree of representative *hsdS* variants from 20 major CCs, selected as described in Materials and Methods. Tip colors indicate CC association. For highly similar variants in distinct CCs, overlapping points are shifted left, outlined by a thin dotted line. Variants in the *v*Saβ island of CC121 genomes are marked by colored asterisks. Shaded regions indicate gene clusters (95% identity threshold) representing the distinct *hsdS* variants of interest. (**B**) Pruned clade from a core SNP phylogeny of CC121 genomes rooted using a reference genome from CC133 (ED133, ASM21031v1). Sequences on very long branches (ED133 and two clades representing highly divergent STs) are not shown. Colored dots at the branch tips correspond to the *hsdS* sequence variants present in *v*Saβ for each genome with white dots indicating the absence of the gene. A triangle is used to represent a single large clade that has been collapsed (containing 179 genomes; 170 with the indicated *hsdS* variant and 9 that are missing the gene), drawn proportionally to the longest branch in the clade. Bootstrap support values (UFBoot) are indicated for each node. (**C**) Genomic map of the *v*Saβ island and downstream genes in the two CC121 genomes of FDAARGOS_21 and sp17 [indicated by asterisks in (B)], showing the replacement of one *hsdS* variant with another from a distinct CC. Arrows indicate the location and orientation of genes, with colors and labels used to highlight genes and other features that are characteristic of *v*Saβ. Vertical blocks between sequences indicate regions of shared similarity (MegaBLAST nucleotide identity), with shading proportional to the level of similarity, as indicated by the scale bar. Variants of *hsdS* are indicated by thick arrow borders and colors according to (A) and (B).

Having experimentally demonstrated the role of LT in mobilizing defense systems and creating hybrid strains with different combinations of immune systems, we analyzed the impact of these processes on natural populations and their potential influence on the evolutionary trajectory of clinical isolates. As previously reported, different CCs typically contain distinct variants of type I R-M systems (*43*), but the extent and distribution of the variation have not been comprehensively examined. We hypothesized that if LT contributes to the transfer of defense systems encoded downstream of phage and PICI *attB* sites, that evidence for HGT events would exist among natural populations. To investigate this further, we examined the variation of the *hsdS* gene in a database of 4000 *S. aureus* genome sequences representing the breadth of species diversity (table S2). Specifically, we searched for CCs that contained variants of *hsdS* that were shared with phylogenetically unrelated CCs, suggesting HGT. Consistent with our in vitro experimental data, we identified CCs with multiple *hsdS* variants that were also identified in unrelated CCs (Fig. 3A). Specifically, CC121, an important global pathogenic clone associated with skin and soft tissue infections, had multiple *hsdS* variants that were shared with other CCs (Fig. 3B). Comparison of the *v*Saβ region in CC121 strains revealed variation in gene content in the region flanking the *hsdS* gene, indicating gene replacement events that could be mediated by LT-mediated transfer followed by recombination (Fig. 3C). In the chimeric strain, another gene mobilized from a different CC, located adjacent to the *hsdS* gene, corresponds to the antiphage Avast type II system. These findings suggest that HGT events in chromosomal regions associated with high levels of LT activity have facilitated the dissemination of not only R-M systems but also defense islands between CCs. The presence of unique combinations of type I R-M system variants across different CCs highlights a crucial role for LT in the emergence and evolution of intraspecies lineages.

### Mobilization of defense islands by LT in *E. coli*

Having confirmed that LT can efficiently mobilize defense systems between *S. aureus* strains, we next investigated whether a similar process could occur in Gram-negative pathogens. It was previously shown that most gains and losses of genes in *Escherichia coli* take place in a limited number of locations called hotspots (*44*). Recently, Hochhauser *et al.* (*45*) analyzed more than 1300 *E. coli* genomes, identifying the chromosomal hotspots with the antiphage systems. Some of these hotspots were associated with classical or uncharacterized MGEs. Others were integrated in a chromosomal region without obvious MGEs. We hypothesized that defense hotspots might be located near prophage integration *attB* sites, which would facilitate their movement via LT.

To test this, we analyzed 2527 complete *E. coli* genomes where we identified 3218 distinct spots (see Materials and Methods). We identified the Ints in the spots to identify potential integration sites and the position of prophages to identify those integration sites occupied by prophages in at least one genome (fig. S5A). While the frequency of prophages varies a lot between hotspots, integration sites with at least one prophage in a genome are found across the chromosome. As a result, almost all the chromosome is at a few capsid headfuls away from the closest integration site (even though this may not be occupied by a prophage in the focal genome). This is further illustration of how LT can potentially mobilize any region of the *E. coli* genome.

We identified 17,829 defense systems in the *E. coli* genomes. Among these, 72.6% are in the spots that have a prophage in at least one chromosome of the species. Many of the remaining defense systems are found in spots containing a very low number of systems, as well as in the loci of MazEF (*46*), which is present in a large fraction of the genomes (86%) (see table S3). Hence, most defense systems are in regions where prophages were shown to be able to integrate.

**Fig. 4.**
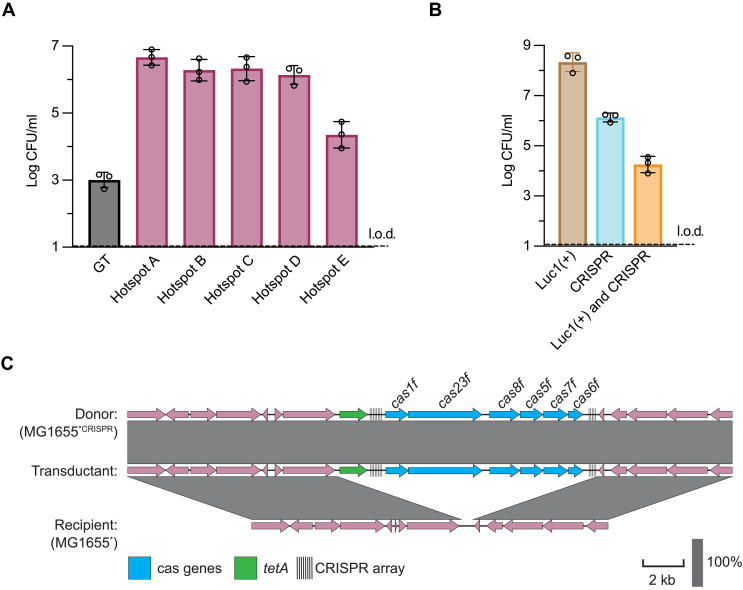
Mobilization of CRISPR-Cas systems via LT in *E. coli*. (**A**) In *E. coli* MG1655*, tetracycline markers were inserted into one of five hotspots (A to E; see Results for details) downstream of the prophage Luc1(+) *attB* site or elsewhere in the chromosome to measure GT. Upon MC induction of Luc1(+), the efficiency of transfer to MG1655* was measured. Log-transformed CFU per milliliter of donor lysate are provided. Each bar represents the means of three biological replicates ± SD. MG1655* represents an MG1655 derivative strain with a deleted R-M (ΔRM) system and restored WbbL expression, enabling production of the O-antigen phage receptor. (**B**) Measuring the efficiency of phage transfer of Luc1(+) (chloramphenicol resistance marker), individual LT transfer of the CRISPR-Cas system (tetracycline resistance marker), and the joint transfer [Luc1(+) and CRISPR] from an MC-induced donor lysogen to the recipient. Log-transformed CFU per milliliter of donor lysate are provided. Each bar represents the means of three biological replicates ± SD. (**C**) Schematic showing the genomic organization surrounding the type I-F CRISR-Cas system in hotspot C (*attB* +120 kb) (top), with the recipient containing an empty hotspot site (bottom). A transductant from (A) was sequenced (middle), showing that the CRISPR-Cas system had been transferred by LT.

To experimentally validate the previous observations, we used phage Luc1+, which integrates into the *E. coli* chromosome upstream of five hotspots identified by Hochhauser *et al.* (hotspots 27 to 31, renamed hotspots A to E). Given that these hotspots are localized next to the Luc1(+) *attB* site, following the directionality of packaging, we hypothesized that Luc1(+) could mobilize these hotspots by LT upon prophage induction. To confirm that Luc1(+) engages in LT, we made an *E. coli* K12 MG1655 derivative strain (named MG1655*) with a deleted R-M system and restored *wbbL* expression, enabling production of the O-antigen phage receptor. Next, we inserted a tetracycline resistance marker (*tetA*) into each of the aforementioned hotspot locations in MG1655*, located ~50, 80, 120, 150, and 260 kilo–base pairs (kbp) downstream of the Luc1(+) *attB* site, respectively, within the expected LT region. As a GT control, we inserted a marker elsewhere in the chromosome (Materials and Methods). These different strains were induced with MC, and the *tetA* transfer efficiencies were measured. As shown in Fig. 4A, all the markers in the hotspots were moved at high frequencies and more efficiently than GT transfer.

Next, we attempted to transfer the complete type I-F CRISPR-Cas adaptive immune system, which is located in hotspot C and present in 5% of our dataset (117 of 2527 strains). This complete system, comprising various *cas* genes and two CRISPR arrays, is about 10 kbp and was inserted into its native location in MG1655*. To track its mobility, we also added a *tetA* marker to the island. Our experiments showed efficient transfer of the marker downstream of Luc1(+) into MG1655*, with rates exceeding that of GT (fig. S5B). The cotransfer of the CRISPR island with the marker was confirmed through PCR analysis of transductants (fig. S5C) and WGS (Fig. 4C). These results indicate that LT plays a critical role in mobilizing defense systems located in hotspots in Gram-negative bacteria. It is evolutionarily intriguing that the phage mobilizing the defense systems could potentially be protected by the CRISPR system it mobilizes.

Last, to confirm that defense genes are mobilized in Gram-negative bacteria in nature, we analyzed recently published metagenomic data from human fecal samples (*47*). For this study, DNA was isolated from virus-like particles and sequenced, and several genomic regions in *Bacteroides uniformis* were reported to show signatures of DNA mobilization by LT. In several mobilized genomic scaffolds, we identified one or more antiphage genes (fig. S6 and table S4), confirming that bacteria can naturally mobilize defense systems via LT.

### LT facilitates the dissemination of immune systems through the generation of chimeric phages

So far, our work has focused on the mobility of chromosomally encoded antiphage systems. Many defense systems, however, are encoded by MGEs, which explains their mobility between bacteria (*48*). In phages, these are “moron” genes (*49*), expressed independently of the phage life cycle and are often encoded in the lysogenic region or at the end of the phage genome, allowing expression independently of the lytic cycle (*50*). While phage genomes are known to be mosaic, the mechanisms by which phages can swap out their accessory moron genes are less well understood. During LT, the first headful is packaged with the right half of the integrated phage DNA, where packaging begins and moron genes are typically located, and the chromosomal region flanking the *attB* site. Given that many such moron genes are located at the extreme end of the prophage, homologous recombination (which would require also homology in the chromosome to the right of the phage) cannot occur between the prophage and an incoming phage. Instead, we wondered whether LT plays a role in facilitating the movement of defense genes between related phages.

**Fig. 5.**
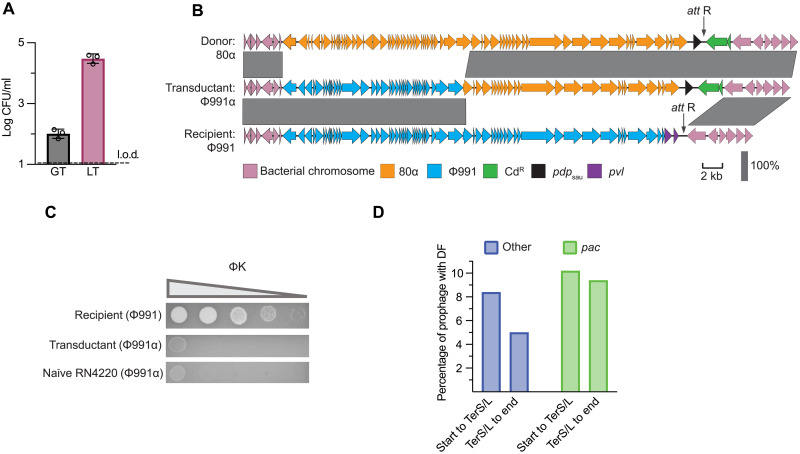
Generation of chimeric phages with new accessory genes through LT. (**A**) The efficiency of LT was assessed by inducing an *S. aureus* RN4220 donor strain harboring prophage 80α and an adjacent chromosomal Cd resistance marker, and that of GT was assessed by using a chromosomal Cd resistance marker elsewhere in the chromosome. Log-transformed CFU per milliliter of donor lysate are provided. Each bar represents the means of three biological replicates ± SD. (**B**) Schematic displaying prophage 80α (top) and Φ991 (bottom) along with the surrounding chromosomal regions. After LT, a transductant from (A) was sequenced, showing the generation of chimeric phage Φ991α. *pvl*, Panton-Valentine leukocidin. (**C**) Phage ΦK was used to infect the original Φ991 recipient and a transductant from (A). In addition, a Φ991α lysogen was MC induced, and the resulting phage was used to lysogenize a naïve (nonlysogenic) RN4220 strain, which was also challenged with phage. Tenfold phage dilutions were spotted, with decreasing phage titer indicated by the triangle. The image is representative of three biological replicates. (**D**) In *E. coli*, a bioinformatic survey assessed the prevalence of defense systems in prophages. Phages were either predicted to use a *pac*-mediated DNA packaging mechanism capable of mediating LT (*pac*) or alternative packaging mechanisms incapable of LT (Other). Defense systems (DF) were categorized depending on their location within the prophage, either from the start of the prophage to *terS*/*L* (does not undergo LT) or from after *terS*/*L* to the end of the prophage (engages in LT).

To test this, we used two prophages, 80α and ϕ991, whose packaging modules (which will be mobilized by LT) are almost identical except for the flanking moron gene(s) (fig. S7A and table S5). 80α carries the *pdp*_Sau_ antiphage gene, an abortive infection system that protects against kayviruses (*51*), while the ϕ991 prophage encodes two genes that comprise the Panton-Valentine leukocidin, an exotoxin. In the donor strain containing 80α, we inserted a Cd marker right downstream of the right attachment site (*attR*) (to ensure the measurement of LT as opposed to full phage transfer). As a GT control, we used a Cd marker elsewhere in the genome (Materials and Methods). Upon MC induction of these strains, we saw high simultaneous transfer of the marker, indicating LT, with significantly lower mobility for the GT marker (Fig. 5A). The transfer of *pdp_Sau_* was confirmed through PCR analysis of transductants (fig. S7B). We performed WGS on a resultant transductant (fig. S7B), revealing a chimeric phage, denoted ϕ991α, where the genome before the phage packaging signal is the preexisting ϕ991, while the packaging region and *pdp_Sau_* are derived from 80α through LT.

Next, we investigated whether the chimeric ϕ991α phage had acquired phage resistance. We used phage K to infect ϕ991 and ϕ991α, which confirmed that the ϕ991α lysogen, but not the original ϕ991 lysogen, could block K infection (Fig. 5C). To ensure that the chimeric ϕ991α phage was fully functional and mobile, we induced the transductant to generate ϕ991α phage particles and used this lysate to lysogenize a naïve RN4220 strain. This transfer was successful, and the lysogenized bacterium retained the phage resistance of the initial transductant (Fig. 5C). In summary, our experiments demonstrate that in addition to mobilizing chromosomally encoded antiphage systems, LT causes diversification of phage genomes, allowing phages to acquire new moron genes, including immune systems.

To extend our previous findings, we analyzed *E. coli* genomes and hypothesized that prophages using the headful packaging mechanism (also known as *pac* phages) should harbor more defense systems in the region between the *pac* site and the end of the prophage (following the directionality of packaging) compared to prophages using the *cos* system. It is important to note that *pac* phages engage in LT, whereas *cos* phages do not. Our results indicate that many defense systems are in the first half of the prophage in the *cos/pac* region, which may or may not be transferred by LT depending on the exact position of these DNA motifs as previously reported for P2-like prophages (*24*). Our results demonstrate that downstream of the cos/*pac* site, *pac* phages contain more defense genes than *cos* phages, likely to allow them to be moved by LT (Fig. 5D).

Last, given that SaPIs also engage in LT (*32*), we hypothesized that many of these elements would carry defense systems and other accessory genes in the region between the SaPI *pac* site and the end of the SaPI genome. This arrangement would allow them to use LT for mobility and to generate SaPI variants. As shown in fig. S7D, this was the case, confirming the important role of LT in driving the generation of prophage and SaPI variants with diverse arsenals of virulence and defense systems.

## DISCUSSION

To defend against phages, bacteria use a myriad of diverse immune systems. A key feature of these antiphage genes is their mobility, being easily gained and lost from populations to quickly adapt to new threats and to prevent fitness costs associated with these genes (*16*, *19*, *21*, *52*, *53*). This mobility is partially explained by the fact that many systems are encoded by MGEs (*24*–*26*), which themselves are mobile. Still, other systems are chromosomally encoded, often in defense islands, and are not inside MGEs (*23*, *54*, *55*). This raises the question of how immune genes and defense islands are mobilized and disseminated in nature.

Here, we show that not only virulence and toxin genes (*27*, *32*, *56*) but also chromosomal antiphage systems can be transferred between bacteria using LT, mediated by phages and PICIs, and LcT, driven by PICIs. During LT, several headfuls of chromosomal DNA are packaged into capsids and released as infective particles, being able to inject DNA into new hosts where it can integrate into the recipient’s chromosome through homologous recombination. Given that several hundred kilo–base pairs of DNA downstream of the phage/PICI can be transferred (*27*, *28*, *32*, *56*), and because phages and PICIs have *attB* sites scattered throughout most bacterial genomes (fig. S5A), a large part of the chromosome, including defense islands containing antiphage systems, can be mobilized by LT. The upper limit of DNA packaged per capsid for most temperate phages is about 45 kbp, meaning that even large defense islands, containing multiple defense genes, can be moved. While our study focused on *S. aureus* and *E. coli*, LT is a hallmark of many temperate phages (*27*–*29*, *32*, *47*), so we anticipate that this strategy for transferring immune genes between bacteria is common in nature. The tendency for antiphage systems to cluster together into defense islands might in part be explained by their ability to be mobilized together in the same phage capsid during LT. Although similar transfer events can occur through GT, as demonstrated for the *Pectobacterium atrosepticum* type I-F CRISPR-Cas system (*57*), previous reports and our data suggest that this is less efficient and therefore likely only plays a relatively minor role.

An intriguing observation from this study is that LT not only facilitates the high transfer of defense islands but also explains how certain defense systems can be lost from subsets of the population. As seen with the LT-mediated transfer of defense islands present in *v*Saα and *v*Saβ, recipient strains replaced some *hsdM*/*hsdS* alleles with others. This highlights the dynamic nature of these systems, underscoring the idea that they can not only be gained but also rapidly lost within bacterial populations.

While the diversification of their immune repertoire is clearly beneficial for the recipient host, does the movement of antiphage genes also benefit the phage or PICI? In the case of LcT, where the PICI genome moves with chromosomal DNA, the PICI is protected by the defense genes it travels with (Fig. 1, D and E). The PICI (and likely phages) can also prime recipients by replacing R-M components with alleles that do not restrict the incoming PICI (Fig. 2E). For phages, LT likely enhances the fitness of potential hosts, ensuring the survival of both the host and phage in the future. The mobility of the immune system via LT can also be used by the phage to combat other MGEs, as previously reported for the mobility of SaPIs encoding immune systems by phages, which could be used as weapons against competitors (*25*). After the transfer, the prophage and the antiphage system coexist in the same cell, and the defense system is unlikely to target its adjacent phage but can inhibit other phages.

We also show that beyond moving chromosomal genes, LT also allows diversification of phage (and probably SaPI) accessory (moron) genes (*49*). While phages are mosaic and are known to frequently exchange DNA, including immune genes, via homologous recombination (*49*, *58*–*60*), it is not clear when and how this exchange occurs. In the first headful of LT, the DNA packaged is both phage DNA and chromosomal DNA, which we show allows efficient recombination (Fig. 5, A and B) to generate new, chimeric phage variants. The right flank of phages often encodes antiphage and virulence genes (Fig. 5D and fig. S7D), and we propose that this location is a variability hotspot because of LT-mediated transfer.

Last, our findings underscore an unexpected role of phages in bacterial evolution. Although *S. aureus* can infect multiple animal species, there is a notable correlation between CCs and their preferred hosts, with many CCs being host-specific (*61*). Different CCs harbor distinct combinations of R-M systems (*41*), which were thought to restrict the exchange of DNA between different CCs. Here, we show that LT and LcT can transfer different MS alleles between CCs to create chimeric strains. These strains have different virulence and defense systems and altered abilities to accept foreign DNA. This raises the intriguing possibility that the mobility of R-M systems via LT or LcT represents an early stage in the formation of new CCs—a process potentially linked to the emergence of novel virulent clones. By analyzing *S. aureus* genomes, we were able to identify such chimeric strains in nature (Fig. 3). Consistent with this, we recently reported that some *S. aureus* strains lack the restriction component of their type I R-M systems (*hsdR*) while still harboring *hsdM*/*hsdS*. These strains act as “gateways” for genetic exchange, where MGEs can obtain methylation and then spread to R-M–competent cells (*62*). Such “gateway” strains, together with the highly efficient LT/LcT-mediated transfer of *hsdM*/*hsdS* described in this study, may help to circumvent inter-CC restriction and promote the evolution of novel strains.

In summary, our work uncovers unexpected roles for LT and LcT, not only as key drivers of antiphage system transfer between bacterial strains but also as major shapers of bacterial population structures. While phages represent a major threat to bacterial populations, they simultaneously enable bacteria to rapidly adapt to challenges—be it phage attacks or colonization of different hosts—by serving as vehicles for defense genes and other auxiliary functions (*63*). This marked duality sheds light on the bacteria-phage arms race, offering fresh insights into how bacterial pathogen populations evolve and adapt. These findings have profound implications for understanding the dynamics of bacterial populations and the emergence of pathogenic strains.

## MATERIALS AND METHODS

### Bacterial strains and growth conditions

All bacterial strains and plasmids used in this study are listed in tables S6 and S7, respectively. The *S. aureus* strain RN4220 and its derivatives were grown in tryptic soy broth (TSB) or TSB agar at 37°C. *E. coli* strains, including MG1655 derivatives, were grown in lysogeny broth (LB) or LB agar at 37°C. Antibiotic selection was performed using erythromycin (10 μg/ml), chloramphenicol (20 μg/ml for *E. coli* and 10 μg/ml for *S. aureus*), tetracycline (20 μg/ml for *E. coli* and 3 μg/ml for *S. aureus*), ampicillin (100 μg/ml), kanamycin (50 μg/ml), or 0.1 mM CdCl_2_ as required. For phage infections, cultures were supplemented with 5 mM CaCl_2_.

### Plasmid construction and genome engineering

For *S. aureus*, construction of plasmids was performed using Gibson assembly (*64*) or restriction-ligation methods. Briefly, for certain constructs, genomic DNA was first amplified with specific primers (indicated in table S8) to produce fragments, which were digested with restriction enzymes. For other constructs, fusion PCR was performed for assembling complex inserts. These fragments were then ligated into the allelic-exchange vectors pBT2-βgal or pMAD-βgal, which allowed the introduction of desired mutations or markers through homologous recombination. Plasmid sequences were verified by Sanger sequencing (Eurofins Genomics). The desired DNA fragments were integrated into the chromosome via temperature-sensitive replication. In this way, selection markers (e.g., chloramphenicol resistance and Cd resistance) were inserted adjacent to genes of interest to facilitate the tracking of LT events.

For *E. coli*, λ Red recombinase–mediated genome engineering was carried out (*65*). Briefly, the PCR product was transformed into the recipient strain harboring plasmid pWRG99, which expresses the λ Red recombinase, and the markers (e.g., tetracycline resistance) were inserted into the bacterial chromosome. The resulting mutants were verified by PCR and Sanger sequencing (Eurofins Genomics).

### Phage and PICI preparation and titration

Phages and PICIs used in this study are listed in table S5. Phages were propagated in appropriate bacterial hosts as described previously (*66*). Phage Luc1(+) was propagated using an MG1655 derivative in which the *wbbL* gene was restored to produce a positive O-antigen phenotype to allow infection. For phage induction, lysogenic strains were grown to an optical density at 600 nm (OD_600_) of 0.25, followed by the addition of MC (2 μg/ml). For SaPI (PICI) induction, SaPI-harboring strains were lysogenized by helper phages (80α^Δ*terS*^ or Φ11) and then induced with MC to trigger SaPI excision and replication. The 80α mutant used carries a mutation in the *terS* gene, which encodes the small terminase subunit essential for phage DNA packaging. This mutation prevents the phage from packaging its own DNA or performing phage-mediated GT and LT while still allowing the transfer of SaPIs and SaPI-mediated LT. Cultures were incubated at 30°C with shaking (80 rpm) until complete lysis occurred (about 4 hours). Phage lysates were filter sterilized using 0.2-μm syringe filters (Sartorius Stedim Biotech) and stored at 4°C.

Phage titration was performed to quantify the number of plaque-forming units in phage lysates. For titration, serial 10-fold dilutions of phage lysates were prepared in phage buffer (PHB). A total of 100 μl of each dilution was mixed with 50 μl of recipient cells (*S. aureus* at OD_540_ = 0.2 or *E. coli* at OD_600_ = 0.2) supplemented with 5 mM CaCl_2_. The mixture was incubated at room temperature for 5 min to allow phage adsorption. Following incubation, 3 ml of top agar was added, and the mixture was poured onto corresponding base agar plates. Plates were incubated at 37°C for 24 hours, and plaques were counted to calculate plaque-forming units per milliliter of the original lysate.

### LT, phage, and SaPI transduction assays

To evaluate LT-mediated mobilization of antiphage systems, and phage or SaPI (PICI) transduction, transduction assays were performed with donor lysates and recipient cells. A total of 1 ml of *S. aureus* recipient cells at an OD_540_ of 1.4 or *E. coli* recipient cells at an OD_600_ of 1.4, supplemented with 5 mM CaCl_2_, was infected with 100 μl of phage lysate diluted to the appropriate concentration in PHB. The infection was allowed to proceed for 30 min at 37°C. The mixture was poured onto selective agar plates containing the appropriate antibiotic to select for transductants. Plates were generally incubated at 37°C for 24 hours. For Cd- or erythromycin-selective plates, incubation times of 36 to 48 hours were required to allow for visible colony formation. Transduction efficiency was determined by counting the resulting colony-forming units (CFU) per milliliter. Data from LT experiments were analyzed using GraphPad Prism.

### Selectable markers and assessment of LT versus GT

Selectable markers were positioned either inside or outside the expected LT region, defined by the *pac* site orientation and previously mapped packaging gradients for each phage or SaPI (*27*, *32*). Markers located within the LT region are copackaged with the phage genome during LT, whereas markers positioned outside this region are transferred exclusively by GT. LT and GT efficiencies were measured using independent donor strains and separately prepared lysates for each marker (fig. S2).

Markers positioned within the LT region were used as LT reporters, whereas those placed outside this region served as GT controls, as previously defined by *pac* orientation and packaging gradients. The chromosomal locations of all selective markers used to distinguish LT from GT in *S. aureus* NCTC 8325 (accession number NC_007795.1) and *E. coli* K-12 MG1655 (accession number NC_000913) are listed in table S9. Primer sequences used to construct and verify each reporter are provided in table S8.

### Unmethylated phage production

To evaluate the functionality of transferred R-M system components (MS_A_ or MS_B_, with different alleles *of hsdM* and *hsdS*) in recipient strains, the production of unmethylated phages was necessary. To generate unmethylated *S. aureus* phages, donor phages were lysogenized in RN4220Δ(MS), a strain in which the methyltransferases (*hsdM_A/B_*) and specificity (*hsdS_A/B_*) genes of the type I R-M system were deleted. These strains were then induced as described above, and the resulting lysates were stored at 4°C. These unmethylated phages were confirmed by spot assays using a restriction-proficient recipient strain to ensure restriction, indicating unmethylated phage DNA.

### Phage methylation by MS_D_

To produce phages methylated by the MS_D_ system, a stepwise method involving plaque isolation and propagation was performed to ensure that the phages acquired the appropriate methylation pattern. Initially, phage lysates were diluted to an appropriate concentration in PHB. Ten microliters of each dilution was spotted onto preprepared phage base agar (PBA) plates containing a lawn of JP9172, a strain encoding the MS_D_ alleles, supplemented with 5 mM CaCl_2_. After incubation at 37°C for 18 to 24 hours, individual plaques were selected using a sterile pipette tip. The isolated plaques were resuspended in 100 μl of PHB and used to infect JP9172 at an OD_540_ of 0.5 in the presence of 5 mM CaCl_2_. After a 10-min adsorption period at 37°C, 3 ml of phage top agar (PTA) was added, and the mixture was poured onto fresh PBA plates. Plates were incubated at 37°C for 24 hours to allow the formation of new plaques. To obtain high-titer methylated phages, the PTA layer containing plaques was scraped off the PBA plates and resuspended in 2 ml of PHB. The mixture was vortexed thoroughly to release the phages into the buffer and then filter sterilized using 0.2-μm syringe filters (Sartorius Stedim Biotech). The resulting phage lysates were stored at 4°C for further experiments.

### Phage spot assays

To confirm the transfer and functionality of mobilized antiphage systems, phage spot assays were conducted. Bacteria grown overnight were first diluted to an OD_540_ of 0.6 before 300 μl of these cells was mixed with 9 ml of PTA and poured onto plates of PBA. These plates were dried at room temperature for 30 min until the PTA had solidified. Phage lysates were serially diluted in 10-fold steps in PHB before 5 μl of each dilution was spotted onto the bacteria lawn and allowed to air-dry. Plates were incubated overnight at 37°C before visualization and imaging.

### WGS and comparative genomic analyses

Genomic DNA was extracted using the GenElute Bacterial Genomic DNA Kit (Sigma-Aldrich) following the manufacturer’s protocol and sequenced on the Illumina NextSeq 500 platform at SeqCenter LLC (US). Reads were quality checked with FastQC, trimmed using Trimmomatic, and processed using the Bacterial & Viral Bioinformatics Resource Center (BV-BRC) platform (www.bv-brc.org/). Assembly was performed with BV-BRC’s integrated pipeline, and annotation was generated using its automated tools. Detailed procedures are described on the BV-BRC website. Genomic comparisons among the donor, recipient, and transductant strains were conducted using Easyfig 2.2.2 (https://mjsull.github.io/Easyfig/) (*67*), which aligned genomic regions of interest and visualized synteny. Conserved and variable regions were highlighted to reveal differences between strains. Figures generated by Easyfig were edited using Adobe Illustrator. Adjustments included layout optimization, annotation enhancement, and highlighting key genomic features such as transduced antiphage systems and their flanking regions. BioRender was used for schematic illustration in fig. S2.

### PCR validation of transductants

To confirm the successful transfer of antiphage systems via LT, a PCR-based approach was used. Transductants were selected from antibiotic-selective plates, and 10 individual colonies were randomly picked for analysis. Colonies were directly used as templates for PCR.

Oligonucleotides used in this study are listed in table S8. These primers targeted regions within the transferred antiphage systems to ensure specificity to the donor sequence. The donor strain served as the positive control, and the recipient strain was used as the negative control to verify the absence of the target sequences in the original recipient genome. For larger systems such as the CRISPR island in *E. coli*, primers were designed to amplify regions at the beginning and end of the CRISPR array to confirm the transfer of the entire system.

PCR products were resolved on a 1.5% agarose gel stained with ethidium bromide and visualized under ultraviolet light. Successful transfer was confirmed by the presence of specific amplicons in transductant samples that matched the donor-positive control but were absent in the recipient-negative control. For CRISPR islands, amplification of both flanking regions validated the complete transfer.

### *S. aureus* genome sequence analysis

We used a database of 4000 previously assembled genomes from 20 major *S. aureus* CCs (1, 121, 130, 133, 151, 188, 15, 22, 30, 45, 5, 7, 8, 9, 59, 77, 88, 93, 398, and 97), including 200 from each. Assemblies were either downloaded from National Center for Biotechnology Information (July 2019) or selected from an in-house collection, chosen to maximize assembly quality, diversity within CCs, and available genome metadata. We used the MLST tool (https://github.com/tseemann/mlst) to calculate STs (sequence types), which were input to the geoBURST algorithm in Phyloviz (ven2.0) (*68*) to determine the CCs. A full list of assemblies, including metadata and quality statistics, is included in table S2. Genes were annotated against the “Staphylococcus” database in PROKKA (version 1.14) (*69*), with default settings for other parameters. To analyze variation in the genomic island *v*Saβ, the region was reannotated in the same way but with known proximal genes given as priority annotations. To detect *hsdS* variants across the data, PIRATE (version 1.04) (*70*) was used to determine orthologous gene clusters at the 50, 70, 90, and 95% protein identity thresholds, specifying the flags to separate the clusters containing paralogs and to retain intermediate data files. For the gene tree, we analyzed a single, large gene family (50% identity) that was found to contain *SauI* variants from both typical genomic locations *v*Saα and *v*Saβ. We used gene clustering at the 95% identity threshold in PIRATE to define *hsdS* variants and selected a representative subset for further analysis that included two gene sequences from each gene cluster at this threshold. Several gene clusters contained sequences from multiple different CCs, and in these cases, we selected two sequences per CC. Representative sequences were selected as the coding DNA sequences (CDSs) with the closest to average length (base pairs), and CDSs with highly truncated CDSs (<700 base pairs) were not included.

### *S. aureus* phylogenetic analysis

Phylogenetic analysis was performed with core single-nucleotide polymorphisms (SNPs) for the 200 CC121 genomes, a CC133 reference outgroup (ED133, ASM21031v1), and a complete CC121 reference genome used to call variants (strain XQ, ASM144434v1). The core SNP alignment was generated with Snippy (version 4.4.5) using Gubbins (version 2.3.4) (*71*) and snp-sites (version 2.5.1) (*72*) to detect and filter out SNPs from recombined regions. A maximum-likelihood phylogeny was then constructed with IQtree (version 2.3.6) (*73*) using the general time reversible model, a discrete four-category gamma distribution to model rate heterogeneity and a correction for ascertainment bias. To provide branch support, we used both the ultrafast bootstrap algorithm with 5000 replicates and an approximate likelihood ratio test. The *hsdS* gene tree was also constructed in IQtree with the general time reversible model but calculated with empirical site frequencies from the alignment and with 100 standard bootstrap replicates. For this, the alignment was generated using mafft (version 7.490) (*74*).

### Identification of spots in *E. coli*

The sequences and annotations of 2527 complete *E. coli* genomes were retrieved from RefSeq GenBank (May 2023). The pangenome was generated using single-linkage clustering with PanACoTA version 1.2.0 (*75*) to define pangenome families, which consist of protein sets sharing at least 80% sequence identity. A pangenome family was classified as a persistent gene family if at least 90% of genomes contained a single, unique representative of the family. Persistent genes were then used to identify the locations of prophages and defense systems. Genomes were partitioned into intervals, defined as the regions delimited by two consecutive persistent genes. Because persistent genes are conserved across most genomes, intervals from different genomes can be grouped on the basis of these shared genes. A spot was defined as a set of such intervals, typically one per genome, flanked by the same two families of persistent genes. It was considered to contain a prophage or a defense system if at least one interval within the spot harbored either a prophage or a defense system. Thus, of the 3218 identified spots, 174 were found to contain prophages, 85 contained defense systems, and 58 contained both (see table S3).

### Identification of *E. coli* prophages

GeNomad version 1.5.2 (*76*) was then used to identify a total of 14,415 temperate phages, integrated into the *E. coli* chromosome as prophages, using default parameters. A total of 13,998 (97%) prophages were localized in distinct intervals, suggesting the existence of a unique attachment site for these intervals. Of these, 9716 prophages have an Int located at one end of the detected element, which allows for the determination of the prophage’s orientation within the genomic sequence. Ints were identified using the PFAM profiles PF00589 for tyrosine recombinases and the pair of profiles PF00239 and PF07508 for serine recombinases (http://pfam.xfam.org/). All the protein profiles were searched using hmmsearch from HMMer suite version 3.3.2 (default parameters) (*77*). Hits were regarded as significant when their *e*-value was smaller than 10^−3^ and their alignment covered at least 50% of the protein profile. The terminase proteins TerS and TerL were identified using a combination of two approaches: (i) leveraging annotations from GenBank files by searching for the keywords “terminase large subunit” or “terminase small subunit” and (ii) performing a blastp search (*78*) against TerS/TerL protein pairs from six known *pac*-phages. For the blastp-based approach, hits were considered significant if their *e*-value was smaller than 10^−5^ and their alignment covered at least 50% of the query sequence. Among the 9716 prophages with an Int, we identified 5961 prophages (61.4%) containing TerS/TerL proteins. To distinguish prophages with TerS/TerL proteins similar to pac-phages, we selected adjacent TerS and TerL hits (within a window of three genes, although they were consistently found just one gene apart) and generated a histogram to determine a threshold for classifying prophages as pac-related on the basis of the observed similarity percentages. Because of the small size of TerS proteins, their similarity can vary widely, sometimes dropping below 30%. On the other hand, TerL proteins, being larger, typically show a similarity exceeding 60%. On the basis of these differences, we established a 60% similarity threshold to classify prophages as Pac-related using the lowest similarity observed in TerL proteins as the defining criterion. Consequently, 1338 pac-related prophages were identified, with TerL proteins exhibiting greater than 60% similarity.

### Identification of defense systems

Defense systems were identified in complete *E. coli* genomes using DefenseFinder version 1.2.0 (with models version 1.2.4) (*15*). A total of 17,829 defense systems were identified, localized within 14,492 distinct intervals, and concentrated in only 85 spots. Two-thirds of the intervals contain only a single defense system, although this number can reach up to seven distinct systems in some intervals.

Given that defense systems can comprise multiple consecutive genes, we determined the central genomic position (the midpoint) for each system. We then calculated the number of defense systems within a 25-kb window downstream of the prophage start (Int start) and end points. We also analyzed the region between Int-TerS/L and TerS/L-end (the prophage end). Last, we compiled the results for 1338 pac-related prophages, i.e., those with an Int and TerL proteins exhibiting more than 60% similarity, and for 4623 other prophages, i.e., those with an Int and TerL proteins showing less than 60% similarity with known TerL pac-type phages (Fig. 5D). For identifying defense genes in *S. aureus*, we used DefenseFinder version 1.2.0 (version 1.2.4) (*15*) and PADLOC (Prokaryotic Antiviral Defence LOCator) (version 2.0.0) (*79*) to identify known and putative defense systems.

## References

[1] H. Georjon, A. Bernheim, The highly diverse antiphage defence systems of bacteria. Nat. Rev. Microbiol. 21, 686–700 (2023). 37460672 10.1038/s41579-023-00934-x

[2] J. T. Rostøl, L. Marraffini, (Ph)ighting phages: How bacteria resist their parasites. Cell Host Microbe 25, 184–194 (2019). 30763533 10.1016/j.chom.2019.01.009 PMC6383810

[3] H. G. Hampton, B. N. J. Watson, P. C. Fineran, The arms race between bacteria and their phage foes. Nature 577, 327–336 (2020). 31942051 10.1038/s41586-019-1894-8

[4] S. Doron, S. Melamed, G. Ofir, A. Leavitt, A. Lopatina, M. Keren, G. Amitai, R. Sorek, Systematic discovery of antiphage defense systems in the microbial pangenome. Science 359, eaar4120 (2018). 29371424 10.1126/science.aar4120 PMC6387622

[5] L. Gao, H. Altae-Tran, F. Böhning, K. S. Makarova, M. Segel, J. L. Schmid-Burgk, J. Koob, Y. I. Wolf, E. V. Koonin, F. Zhang, Diverse enzymatic activities mediate antiviral immunity in prokaryotes. Science 369, 1077–1084 (2020). 32855333 10.1126/science.aba0372 PMC7985843

[6] C. N. Vassallo, C. R. Doering, M. L. Littlehale, G. I. C. Teodoro, M. T. Laub, A functional selection reveals previously undetected anti-phage defence systems in the *E. coli* pangenome. Nat. Microbiol. 7, 1568–1579 (2022). 36123438 10.1038/s41564-022-01219-4 PMC9519451

[7] A. Millman, S. Melamed, A. Leavitt, S. Doron, A. Bernheim, J. Hör, J. Garb, N. Bechon, A. Brandis, A. Lopatina, G. Ofir, D. Hochhauser, A. Stokar-Avihail, N. Tal, S. Sharir, M. Voichek, Z. Erez, J. L. M. Ferrer, D. Dar, A. Kacen, G. Amitai, R. Sorek, An expanded arsenal of immune systems that protect bacteria from phages. Cell Host Microbe 30, 1556–1569.e5 (2022). 36302390 10.1016/j.chom.2022.09.017

[8] M. R. Tock, D. T. Dryden, The biology of restriction and anti-restriction. Curr. Opin. Microbiol. 8, 466–472 (2005). 15979932 10.1016/j.mib.2005.06.003

[9] R. Barrangou, C. Fremaux, H. Deveau, M. Richards, P. Boyaval, S. Moineau, D. A. Romero, P. Horvath, CRISPR provides acquired resistance against viruses in prokaryotes. Science 315, 1709–1712 (2007). 17379808 10.1126/science.1138140

[10] L. A. Marraffini, E. J. Sontheimer, CRISPR interference limits horizontal gene transfer in Staphylococci by targeting DNA. Science 322, 1843–1845 (2008). 19095942 10.1126/science.1165771 PMC2695655

[11] S. van Houte, A. Buckling, E. R. Westra, Evolutionary ecology of prokaryotic immune mechanisms. Microbiol. Mol. Biol. Rev. 80, 745–763 (2016). 27412881 10.1128/MMBR.00011-16 PMC4981670

[12] A. Chevallereau, B. J. Pons, S. van Houte, E. R. Westra, Interactions between bacterial and phage communities in natural environments. Nat. Rev. Microbiol. 20, 49–62 (2022). 34373631 10.1038/s41579-021-00602-y

[13] P. Gómez, A. Buckling, Bacteria-phage antagonistic coevolution in soil. Science 332, 106–109 (2011). 21454789 10.1126/science.1198767

[14] D. W. Adams, M. Jaskólska, A. Lemopoulos, S. Stutzmann, L. Righi, L. Bader, M. Blokesch, Diverse phage defence systems define West African South American pandemic *Vibrio cholerae*. bioRxiv 624991 [Preprint] (2024). 10.1101/2024.11.23.624991. PMC12137116 40404828

[15] F. Tesson, A. Hervé, E. Mordret, M. Touchon, C. d’Humières, J. Cury, A. Bernheim, Systematic and quantitative view of the antiviral arsenal of prokaryotes. Nat. Commun. 13, 2561 (2022). 35538097 10.1038/s41467-022-30269-9 PMC9090908

[16] E. V. Koonin, K. S. Makarova, Y. I. Wolf, Evolutionary genomics of defense systems in archaea and bacteria. Annu. Rev. Microbiol. 71, 233–261 (2017). 28657885 10.1146/annurev-micro-090816-093830 PMC5898197

[17] P. Maguin, A. Varble, J. W. Modell, L. A. Marraffini, Cleavage of viral DNA by restriction endonucleases stimulates the type II CRISPR-Cas immune response. Mol. Cell 82, 907–919.e7 (2022). 35134339 10.1016/j.molcel.2022.01.012 PMC8900293

[18] Y. Wu, S. K. Garushyants, A. van den Hurk, C. Aparicio-Maldonado, S. K. Kushwaha, C. M. King, Y. Ou, T. C. Todeschini, M. R. J. Clokie, A. D. Millard, Y. E. Gençay, E. V. Koonin, F. L. Nobrega, Bacterial defense systems exhibit synergistic anti-phage activity. Cell Host Microbe 32, 557–572.e6 (2024). 38402614 10.1016/j.chom.2024.01.015 PMC11009048

[19] K. S. Makarova, Y. I. Wolf, S. Snir, E. V. Koonin, Defense islands in bacterial and archaeal genomes and prediction of novel defense systems. J. Bacteriol. 193, 6039–6056 (2011). 21908672 10.1128/JB.05535-11 PMC3194920

[20] K. S. Makarova, Y. I. Wolf, E. V. Koonin, Comparative genomics of defense systems in archaea and bacteria. Nucleic Acids Res. 41, 4360–4377 (2013). 23470997 10.1093/nar/gkt157 PMC3632139

[21] A. Bernheim, R. Sorek, The pan-immune system of bacteria: Antiviral defence as a community resource. Nat. Rev. Microbiol. 18, 113–119 (2020). 31695182 10.1038/s41579-019-0278-2

[22] J. Iranzo, J. A. Cuesta, S. Manrubia, M. I. Katsnelson, E. V. Koonin, Disentangling the effects of selection and loss bias on gene dynamics. Proc. Natl. Acad. Sci. U.S.A. 114, E5616–E5624 (2017). 28652353 10.1073/pnas.1704925114 PMC5514749

[23] F. A. Hussain, J. Dubert, J. Elsherbini, M. Murphy, D. VanInsberghe, P. Arevalo, K. Kauffman, B. K. Rodino-Janeiro, H. Gavin, A. Gomez, A. Lopatina, F. L. Roux, M. F. Polz, Rapid evolutionary turnover of mobile genetic elements drives bacterial resistance to phages. Science 374, 488–492 (2021). 34672730 10.1126/science.abb1083

[24] F. Rousset, F. Depardieu, S. Miele, J. Dowding, A.-L. Laval, E. Lieberman, D. Garry, E. P. C. Rocha, A. Bernheim, D. Bikard, Phages and their satellites encode hotspots of antiviral systems. Cell Host Microbe 30, 740–753.e5 (2022). 35316646 10.1016/j.chom.2022.02.018 PMC9122126

[25] A. Fillol-Salom, J. T. Rostøl, A. D. Ojiogu, J. Chen, G. Douce, S. Humphrey, J. R. Penadés, Bacteriophages benefit from mobilizing pathogenicity islands encoding immune systems against competitors. Cell 185, 3248–3262.e20 (2022). 35985290 10.1016/j.cell.2022.07.014

[26] J. D. Bouchard, E. Dion, F. Bissonnette, S. Moineau, Characterization of the two-component abortive phage infection mechanism AbiT from *Lactococcus lactis*. J. Bacteriol. 184, 6325–6332 (2002). 12399502 10.1128/JB.184.22.6325-6332.2002 PMC151939

[27] J. Chen, N. Quiles-Puchalt, Y. N. Chiang, R. Bacigalupe, A. Fillol-Salom, M. S. J. Chee, J. R. Fitzgerald, J. R. Penadés, Genome hypermobility by lateral transduction. Science 362, 207–212 (2018). 30309949 10.1126/science.aat5867

[28] A. Fillol-Salom, R. Bacigalupe, S. Humphrey, Y. N. Chiang, J. Chen, J. R. Penadés, Lateral transduction is inherent to the life cycle of the archetypical Salmonella phage P22. Nat. Commun. 12, 6510 (2021). 34751192 10.1038/s41467-021-26520-4 PMC8575938

[29] Z. Liu, K. Tang, Y. Zhou, T. Liu, Y. Guo, D. Wu, X. Wang, Active prophages in coral-associated *Halomonas* capable of lateral transduction. ISME J. 18, wrae085 (2024). 38739683 10.1093/ismejo/wrae085 PMC11131426

[30] M. Kleiner, B. Bushnell, K. E. Sanderson, L. V. Hooper, B. A. Duerkop, Transductomics: Sequencing-based detection and analysis of transduced DNA in pure cultures and microbial communities. Microbiome 8, 158–117 (2020). 33190645 10.1186/s40168-020-00935-5 PMC7667829

[31] P. Misson, E. Bruder, J. K. Cornuault, M. D. Paepe, P. Nicolas, G. Demarre, G. Lakisic, M.-A. Petit, O. Espeli, F. Lecointe, Phage production is blocked in the adherent-invasive *Escherichia coli* LF82 upon macrophage infection. PLOS Pathog. 19, e1011127 (2023). 36730457 10.1371/journal.ppat.1011127 PMC9928086

[32] M. S. J. Chee, E. Serrano, Y. N. Chiang, J. Harling-Lee, R. Man, R. Bacigalupe, J. R. Fitzgerald, J. R. Penadés, J. Chen, Dual pathogenicity island transfer by piggybacking lateral transduction. Cell 186, 3414–3426.e16 (2023). 37541198 10.1016/j.cell.2023.07.001

[33] R. P. Novick, Pathogenicity islands and their role in Staphylococcal biology. Microbiol. Spectr. 7, 10.1128/microbiolspec.gpp3-0062-2019 (2019). 10.1128/microbiolspec.gpp3-0062-2019 PMC11257176 31172913

[34] H. Ingmer, D. Gerlach, C. Wolz, Temperate phages of *Staphylococcus aureus*. Microbiol. Spectr. 7, 10.1128/microbiolspec.gpp3-0058-2018 (2019). 10.1128/microbiolspec.gpp3-0058-2018 PMC10921950 31562736

[35] N. Firth, S. O. Jensen, S. M. Kwong, R. A. Skurray, J. P. Ramsay, Staphylococcal plasmids, transposable and integrative elements. Microbiol. Spectr. 6, 10.1128/microbiolspec.gpp3-0030-2018 (2018). 10.1128/microbiolspec.gpp3-0030-2018 PMC11633639 30547857

[36] J. A. Lindsay, A. Ruzin, H. F. Ross, N. Kurepina, R. P. Novick, The gene for toxic shock toxin is carried by a family of mobile pathogenicity islands in *Staphylococcus aureus*. Mol. Microbiol. 29, 527–543 (1998). 9720870 10.1046/j.1365-2958.1998.00947.x

[37] L. P. Cooper, G. A. Roberts, J. H. White, Y. A. Luyten, E. K. M. Bower, R. D. Morgan, R. J. Roberts, J. A. Lindsay, D. T. F. Dryden, DNA target recognition domains in the Type I restriction and modification systems of *Staphylococcus aureus*. Nucleic Acids Res. 45, 3395–3406 (2017). 28180279 10.1093/nar/gkx067 PMC5399793

[38] D. Nair, G. Memmi, D. Hernandez, J. Bard, M. Beaume, S. Gill, P. Francois, A. L. Cheung, Whole-genome sequencing of *Staphylococcus aureus* strain RN4220, a key laboratory strain used in virulence research, identifies mutations that affect not only virulence factors but also the fitness of the strain. J. Bacteriol. 193, 2332–2335 (2011). 21378186 10.1128/JB.00027-11 PMC3133102

[39] N. E. Murray, Type I restriction systems: Sophisticated molecular machines (a Legacy of Bertani and Weigle). Microbiol. Mol. Biol. Rev. 64, 412–434 (2000). 10839821 10.1128/mmbr.64.2.412-434.2000 PMC98998

[40] J. R. Fitzgerald, M. T. G. Holden, Genomics of natural populations of *Staphylococcus aureus*. Annu. Rev. Microbiol. 70, 459–478 (2016). 27482738 10.1146/annurev-micro-102215-095547

[41] D. E. Waldron, J. A. Lindsay, Sau1: A novel lineage-specific type I restriction-modification system that blocks horizontal gene transfer into *Staphylococcus aureus* and between *S. aureus* isolates of different lineages. J. Bacteriol. 188, 5578–5585 (2006). 16855248 10.1128/JB.00418-06 PMC1540015

[42] A. Fillol-Salom, L. Miguel-Romero, A. Marina, J. Chen, J. R. Penadés, Beyond the CRISPR-Cas safeguard: PICI-encoded innate immune systems protect bacteria from bacteriophage predation. Curr. Opin. Microbiol. 56, 52–58 (2020). 32653777 10.1016/j.mib.2020.06.002

[43] A. G. Moller, J. A. Lindsay, T. D. Read, Determinants of phage host range in *Staphylococcus* species. Appl. Environ. Microbiol. 85, e00209–e00219 (2019). 30902858 10.1128/AEM.00209-19 PMC6532042

[44] M. Touchon, C. Hoede, O. Tenaillon, V. Barbe, S. Baeriswyl, P. Bidet, E. Bingen, S. Bonacorsi, C. Bouchier, O. Bouvet, A. Calteau, H. Chiapello, O. Clermont, S. Cruveiller, A. Danchin, M. Diard, C. Dossat, M. E. Karoui, E. Frapy, L. Garry, J. M. Ghigo, A. M. Gilles, J. Johnson, C. L. Bouguénec, M. Lescat, S. Mangenot, V. Martinez-Jéhanne, I. Matic, X. Nassif, S. Oztas, M. A. Petit, C. Pichon, Z. Rouy, C. S. Ruf, D. Schneider, J. Tourret, B. Vacherie, D. Vallenet, C. Médigue, E. P. C. Rocha, E. Denamur, Organised genome dynamics in the *Escherichia coli* species results in highly diverse adaptive paths. PLOS Genet. 5, e1000344 (2009). 19165319 10.1371/journal.pgen.1000344 PMC2617782

[45] D. Hochhauser, A. Millman, R. Sorek, The defense island repertoire of the *Escherichia coli* pan-genome. PLOS Genet. 19, e1010694 (2023). 37023146 10.1371/journal.pgen.1010694 PMC10121019

[46] N. Nikolic, T. Bergmiller, M. Pleška, C. C. Guet, Bacterial toxin-antitoxin system MazEF as a native defense mechanism against RNA phages in *Escherichia coli*. bioRxiv 526697 [Preprint] (2023). 10.1101/2023.02.01.526697.

[47] T. Borodovich, J. S. Wilson, P. Bardy, M. Smith, C. Hill, E. V. Khokhlova, B. Govi, P. C. M. Fogg, C. Hill, A. N. Shkoporov, Large scale capsid-mediated mobilisation of bacterial genomic DNA in the gut microbiome. bioRxiv 623857 [Preprint] (2024). 10.1101/2024.11.15.623857. PMC12946183 41593069

[48] B. Beamud, F. Benz, D. Bikard, Going viral: The role of mobile genetic elements in bacterial immunity. Cell Host Microbe 32, 804–819 (2024). 38870898 10.1016/j.chom.2024.05.017

[49] R. J. Juhala, M. E. Ford, R. L. Duda, A. Youlton, G. F. Hatfull, R. W. Hendrix, Genomic sequences of bacteriophages HK97 and HK022: Pervasive genetic mosaicism in the lambdoid bacteriophages. J. Mol. Biol. 299, 27–51 (2000). 10860721 10.1006/jmbi.2000.3729

[50] J. T. Rostøl, N. Quiles-Puchalt, P. Iturbe-Sanz, Í. Lasa, J. R. Penadés, Bacteriophages avoid autoimmunity from cognate immune systems as an intrinsic part of their life cycles. Nat. Microbiol. 9, 1312–1324 (2024). 38565896 10.1038/s41564-024-01661-6 PMC11087260

[51] L. Kuntová, I. Mašlaňová, R. Obořilová, H. Šimečková, A. Finstrlová, P. Bárdy, M. Šiborová, L. Troianovska, T. Botka, P. Gintar, O. Šedo, Z. Farka, J. Doškař, R. Pantůček, *Staphylococcus aureus* prophage-encoded protein causes abortive infection and provides population immunity against kayviruses. mBio 14, e0249022 (2023). 36779718 10.1128/mbio.02490-22 PMC10127798

[52] J. S. Godde, A. Bickerton, The repetitive DNA elements called CRISPRs and their associated genes: Evidence of horizontal transfer among prokaryotes. J. Mol. Evol. 62, 718–729 (2006). 16612537 10.1007/s00239-005-0223-z

[53] P. H. Oliveira, M. Touchon, E. P. C. Rocha, The interplay of restriction-modification systems with mobile genetic elements and their prokaryotic hosts. Nucleic Acids Res. 42, 10618–10631 (2014). 25120263 10.1093/nar/gku734 PMC4176335

[54] M. C. Johnson, E. Laderman, E. Huiting, C. Zhang, A. Davidson, J. Bondy-Denomy, Core defense hotspots within *Pseudomonas aeruginosa* are a consistent and rich source of anti-phage defense systems. Nucleic Acids Res. 51, 4995–5005 (2023). 37140042 10.1093/nar/gkad317 PMC10250203

[55] S. K. Kushwaha, Y. Wu, H. L. Avila, A. Anand, T. Sicheritz-Pontén, A. Millard, S. A. Marathe, F. L. Nobrega, Comprehensive blueprint of Salmonella genomic plasticity identifies hotspots for pathogenicity genes. PLOS Biol. 22, e3002746 (2024). 39110680 10.1371/journal.pbio.3002746 PMC11305592

[56] S. Humphrey, A. Fillol-Salom, N. Quiles-Puchalt, R. Ibarra-Chávez, A. F. Haag, J. Chen, J. R. Penadés, Bacterial chromosomal mobility via lateral transduction exceeds that of classical mobile genetic elements. Nat. Commun. 12, 6509 (2021). 34750368 10.1038/s41467-021-26004-5 PMC8575950

[57] B. N. J. Watson, R. H. J. Staals, P. C. Fineran, CRISPR-Cas-mediated phage resistance enhances horizontal gene transfer by transduction. mBio 9, e02406–e02417 (2018). 29440578 10.1128/mBio.02406-17 PMC5821089

[58] R. Pinilla-Redondo, S. Shehreen, N. D. Marino, R. D. Fagerlund, C. M. Brown, S. J. Sørensen, P. C. Fineran, J. Bondy-Denomy, Discovery of multiple anti-CRISPRs highlights anti-defense gene clustering in mobile genetic elements. Nat. Commun. 11, 5652 (2020). 33159058 10.1038/s41467-020-19415-3 PMC7648647

[59] H. T. Mohammed, C. Mageeney, J. Korenberg, L. Graham, V. C. Ware, Characterization of novel recombinant mycobacteriophages derived from homologous recombination between two temperate phages. G3 13, jkad210 (2023). 37713616 10.1093/g3journal/jkad210 PMC10700106

[60] M. B. Dion, F. Oechslin, S. Moineau, Phage diversity, genomics and phylogeny. Nat. Rev. Microbiol. 18, 125–138 (2020). 32015529 10.1038/s41579-019-0311-5

[61] A. F. Haag, J. R. Fitzgerald, J. R. Penadés, *Staphylococcus aureus* in animals. Microbiol. Spectr. 7, 10.1128/microbiolspec.gpp3-0060-2019 (2019). 10.1128/microbiolspec.gpp3-0060-2019 PMC11257167 31124433

[62] W. Figueroa, A. Sabnis, R. Ibarra-Chávez, J. Gorzynski, J. R. Fitzgerald, J. R. Penadés, Immune-deficient bacteria serve as gateways to genetic exchange and microbial evolution. bioRxiv 663135 [Preprint] (2025). 10.1101/2025.07.04.663135. PMC13216270 41922341

[63] H. Brüssow, C. Canchaya, W.-D. Hardt, Phages and the evolution of bacterial pathogens: From genomic rearrangements to lysogenic conversion. Microbiol. Mol. Biol. Rev. 68, 560–602 (2004). 15353570 10.1128/MMBR.68.3.560-602.2004 PMC515249

[64] D. G. Gibson, L. Young, R.-Y. Chuang, J. C. Venter, C. A. Hutchison, H. O. Smith, Enzymatic assembly of DNA molecules up to several hundred kilobases. Nat. Methods 6, 343–345 (2009). 19363495 10.1038/nmeth.1318

[65] M. Juhas, J. W. Ajioka, Lambda Red recombinase-mediated integration of the high molecular weight DNA into the *Escherichia coli* chromosome. Microb. Cell Fact. 15, 172 (2016). 27716307 10.1186/s12934-016-0571-y PMC5050610

[66] M. Á. Tormo-Más, J. Donderis, M. García-Caballer, A. Alt, I. Mir-Sanchis, A. Marina, J. R. Penadés, Phage dUTPases control transfer of virulence genes by a proto-oncogenic G protein-like mechanism. Mol. Cell 49, 947–958 (2013). 23333307 10.1016/j.molcel.2012.12.013

[67] M. J. Sullivan, N. K. Petty, S. A. Beatson, Easyfig: A genome comparison visualizer. Bioinformatics 27, 1009–1010 (2011). 21278367 10.1093/bioinformatics/btr039 PMC3065679

[68] M. Nascimento, A. Sousa, M. Ramirez, A. P. Francisco, J. A. Carriço, C. Vaz, PHYLOViZ 2.0: Providing scalable data integration and visualization for multiple phylogenetic inference methods. Bioinformatics 33, 128–129 (2017). 27605102 10.1093/bioinformatics/btw582

[69] T. Seemann, Prokka: Rapid prokaryotic genome annotation. Bioinformatics 30, 2068–2069 (2014). 24642063 10.1093/bioinformatics/btu153

[70] S. C. Bayliss, H. A. Thorpe, N. M. Coyle, S. K. Sheppard, E. J. Feil, PIRATE: A fast and scalable pangenomics toolbox for clustering diverged orthologues in bacteria. GigaScience 8, giz119 (2019). 31598686 10.1093/gigascience/giz119 PMC6785682

[71] N. J. Croucher, A. J. Page, T. R. Connor, A. J. Delaney, J. A. Keane, S. D. Bentley, J. Parkhill, S. R. Harris, Rapid phylogenetic analysis of large samples of recombinant bacterial whole genome sequences using Gubbins. Nucleic Acids Res. 43, e15 (2015). 25414349 10.1093/nar/gku1196 PMC4330336

[72] A. J. Page, B. Taylor, A. J. Delaney, J. Soares, T. Seemann, J. A. Keane, S. R. Harris, SNP-sites: Rapid efficient extraction of SNPs from multi-FASTA alignments. Microb. Genom. 2, e000056 (2016). 28348851 10.1099/mgen.0.000056 PMC5320690

[73] B. Q. Minh, H. A. Schmidt, O. Chernomor, D. Schrempf, M. D. Woodhams, A. von Haeseler, R. Lanfear, IQ-TREE 2: New models and efficient methods for phylogenetic inference in the genomic era. Mol. Biol. Evol. 37, 1530–1534 (2020). 32011700 10.1093/molbev/msaa015 PMC7182206

[74] K. Katoh, D. M. Standley, MAFFT multiple sequence alignment software version 7: Improvements in performance and usability. Mol. Biol. Evol. 30, 772–780 (2013). 23329690 10.1093/molbev/mst010 PMC3603318

[75] A. Perrin, E. P. C. Rocha, PanACoTA: A modular tool for massive microbial comparative genomics. NAR Genom. Bioinform. 3, lqaa106 (2021). 33575648 10.1093/nargab/lqaa106 PMC7803007

[76] A. P. Camargo, S. Roux, F. Schulz, M. Babinski, Y. Xu, B. Hu, P. S. G. Chain, S. Nayfach, N. C. Kyrpides, Identification of mobile genetic elements with geNomad. Nat. Biotechnol. 42, 1303–1312 (2024). 37735266 10.1038/s41587-023-01953-y PMC11324519

[77] S. R. Eddy, Accelerated profile HMM searches. PLOS Comput. Biol. 7, e1002195 (2011). 22039361 10.1371/journal.pcbi.1002195 PMC3197634

[78] S. F. Altschul, T. L. Madden, A. A. Schäffer, J. Zhang, Z. Zhang, W. Miller, D. J. Lipman, Gapped BLAST and PSI-BLAST: A new generation of protein database search programs. Nucleic Acids Res. 25, 3389–3402 (1997). 9254694 10.1093/nar/25.17.3389 PMC146917

[79] L. J. Payne, S. Meaden, M. R. Mestre, C. Palmer, N. Toro, P. C. Fineran, S. A. Jackson, PADLOC: A web server for the identification of antiviral defence systems in microbial genomes. Nucleic Acids Res. 50, W541–W550 (2022). 35639517 10.1093/nar/gkac400 PMC9252829

