# Supplementary material for: Bacteriophages mobilize bacterial defense systems via lateral transduction
Source: Sci Adv. 2026 Jan 23;12(4):eadx5749. doi: 10.1126/sciadv.adx5749 (PMC12829572; doi:10.1126/sciadv.adx5749)
Supplement: Supplementary file 1 — Figs. S1 to S7 Legends for tables S1 to S9 [file sciadv.adx5749_sm.pdf]

Science Advances
AAAS

Supplementary Materials for

Bacteriophages mobilize bacterial defense systems via lateral transduction

Xu Kuang *et al.*

Corresponding author: Jakob T. Rostøl, j.rostoel@imperial.ac.uk; José R. Penadés, j.penades@imperial.ac.uk

Sci. Adv. **12**, eadx5749 (2026)
DOI: 10.1126/sciadv.adx5749

The PDF file includes:

Figs. S1 to S7
Legends for tables S1 to S9

Other Supplementary Material for this manuscript includes the following:

Tables S1 to S9

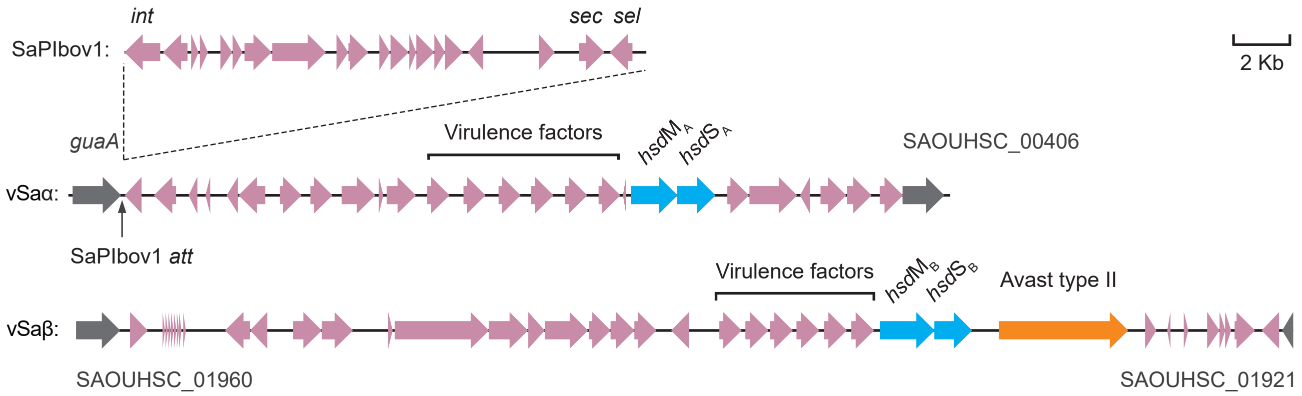

Figure S1. Genomic organization of SaPIbov1, vSaα, and vSaβ in *S. aureus* RN4220. The vSaα genomic island spans the region from *guaA* to SAOUHSC_00406 (accession number NC_007795.1), and vSaβ extends from SAOUHSC_01960 to SAOUHSC_01921. The dashed lines indicate the attachment (*att*) site where SaPIbov1 can integrate into vSaα. The SaPIbov1 element shown above depicts its gene organization and its relative position to vSaα. Genes encoding virulence factors are shown in pink, RM system genes (*hsdM*/*hsdS*) in blue, and the Avast type II system in orange.

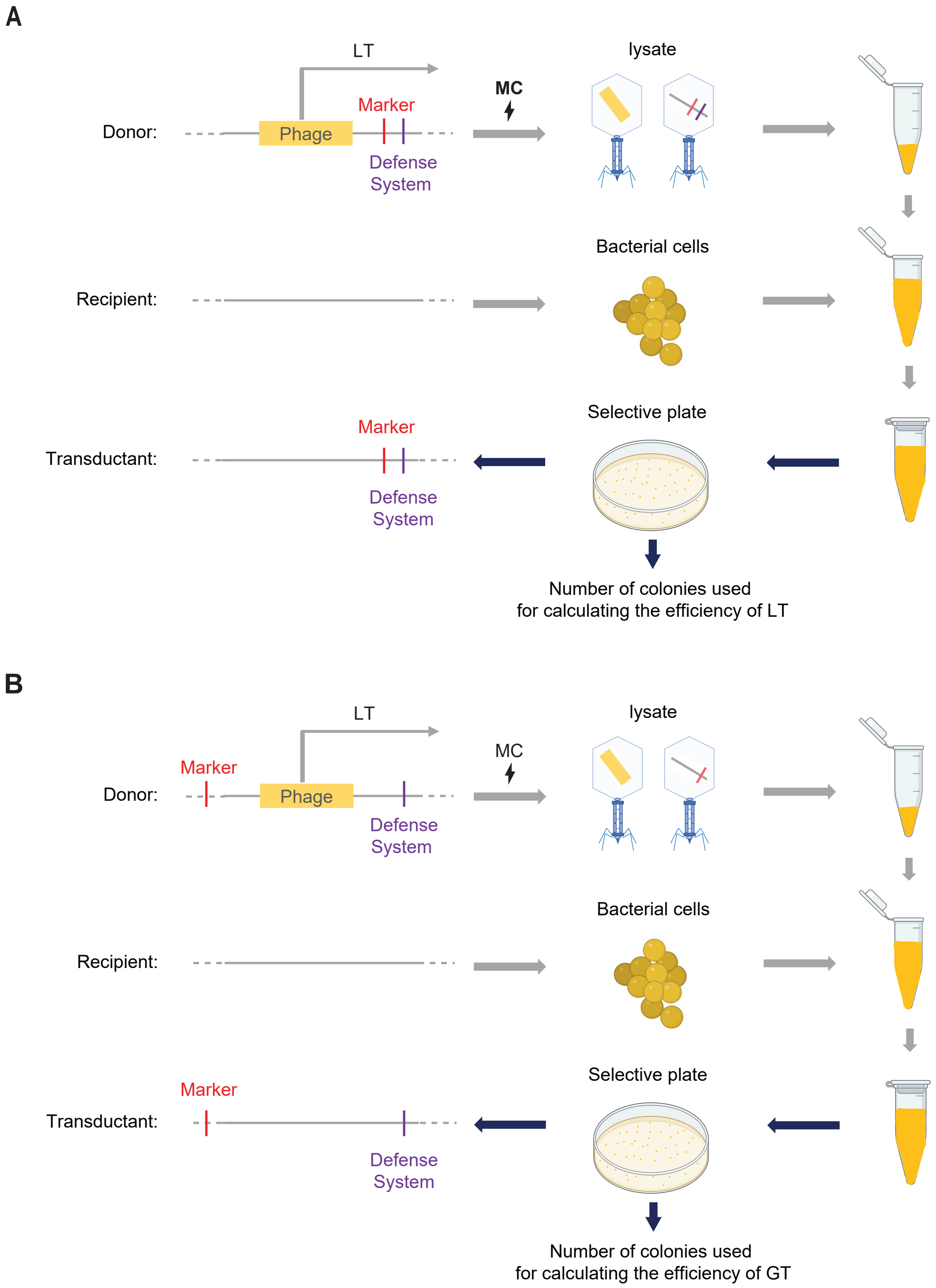

Figure S2. Schematics showing the experimental setups for detecting lateral transduction (LT) (A) and generalised transduction (GT) (B). Schematic illustrating the experimental workflow used to measure the efficiency of LT (A) and GT (B). In both assays, the dashed line indicates the position of the SaPI/phage attachment site (*att*) within the donor cell chromosome. For LT, the prophage/SaPI is integrated upstream of the marker, being packaged by LT when the prophage/SaPI is induced by mitomycin C (MC). For GT, the marker is located upstream of the *att* site, only being transferred by GT when random chromosomal DNA is packaged. Recipient bacteria are then infected with the corresponding lysates, and transductants are selected on antibiotic plates. The number of resulting colonies is used to calculate the efficiencies of LT or GT.

Created in BioRender. Kuang, X. (2026) https://BioRender.com/flryllg

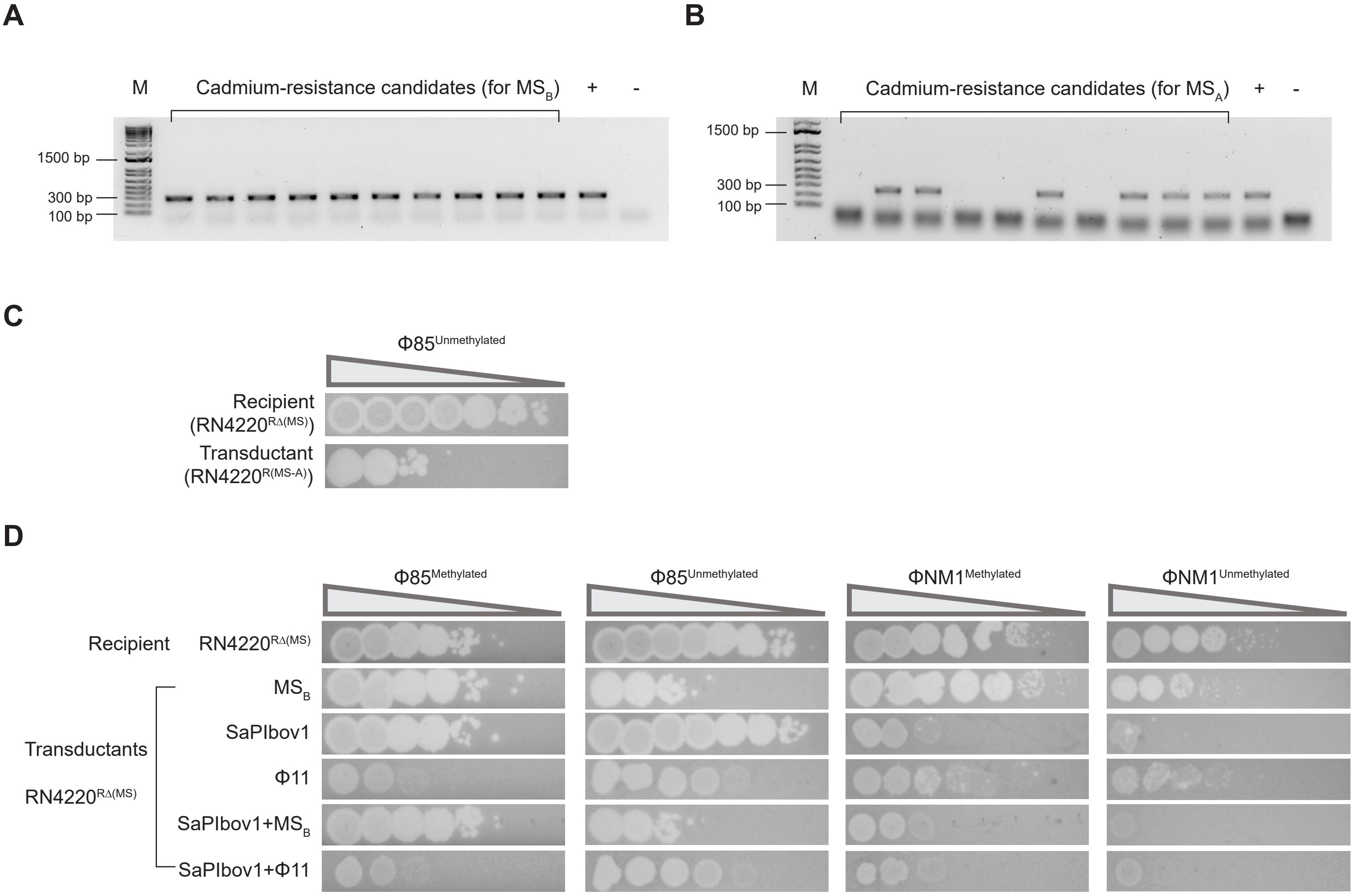

Figure S3. Movement of R-M components by LT. Polymerase chain reactions (PCR) confirming the presence of genes MS$_B$ (**A**) or MS$_A$ (**B**) in cadmium-resistant transductants from Fig. 1(B) indicating successful co-transfer of these genes with the marker. Out of ten colonies for each, all ten were positive for MS$_{(B)}$ and six were positive for MS$_A$, the latter having lower efficiency due to the cadmium resistance marker being located further away from MS$_A$. The PCR products were visualised by agarose gel electrophoresis. Positive (+) and negative (−) controls were included. M: DNA marker. **(C)** Phage resistance of transductants from Fig. 1b carrying MS$_A$. Spot assay with unmethylated phage Φ85 demonstrates enhanced resistance in the transductant (RN4220R$^{(MS\text{-}A)}$) compared to the recipient strain (RN4220$^{R\Delta(MS)}$). **(D)** Assaying the phage resistance profiles of various single or double transductants from Fig. 1f. The defence components MS$_B$ (LT), SaPIbov1, and Φ11 were mobilised from a donor strain in a single induction event and transferred to a recipient, and challenged with unmethylated or methylated Φ85 (non-helper phage) or ΦNM1 (SaPIbov1 helper phage). Overall, MS$_B$ protects against the unmethylated phages, SaPIbov1 against the helper phage ΦNM1, and Φ11 against Φ85 (weakly) and ΦNM1. For the double transductants (SaPIbov1+MS$_B$) and SaPIbov1+Φ11, the protection is stronger than for the single transductants. For **C** and **D**, ten-fold phage dilutions were spotted, with decreasing phage titre indicated by the triangle. Each image is representative of three biological replicates.

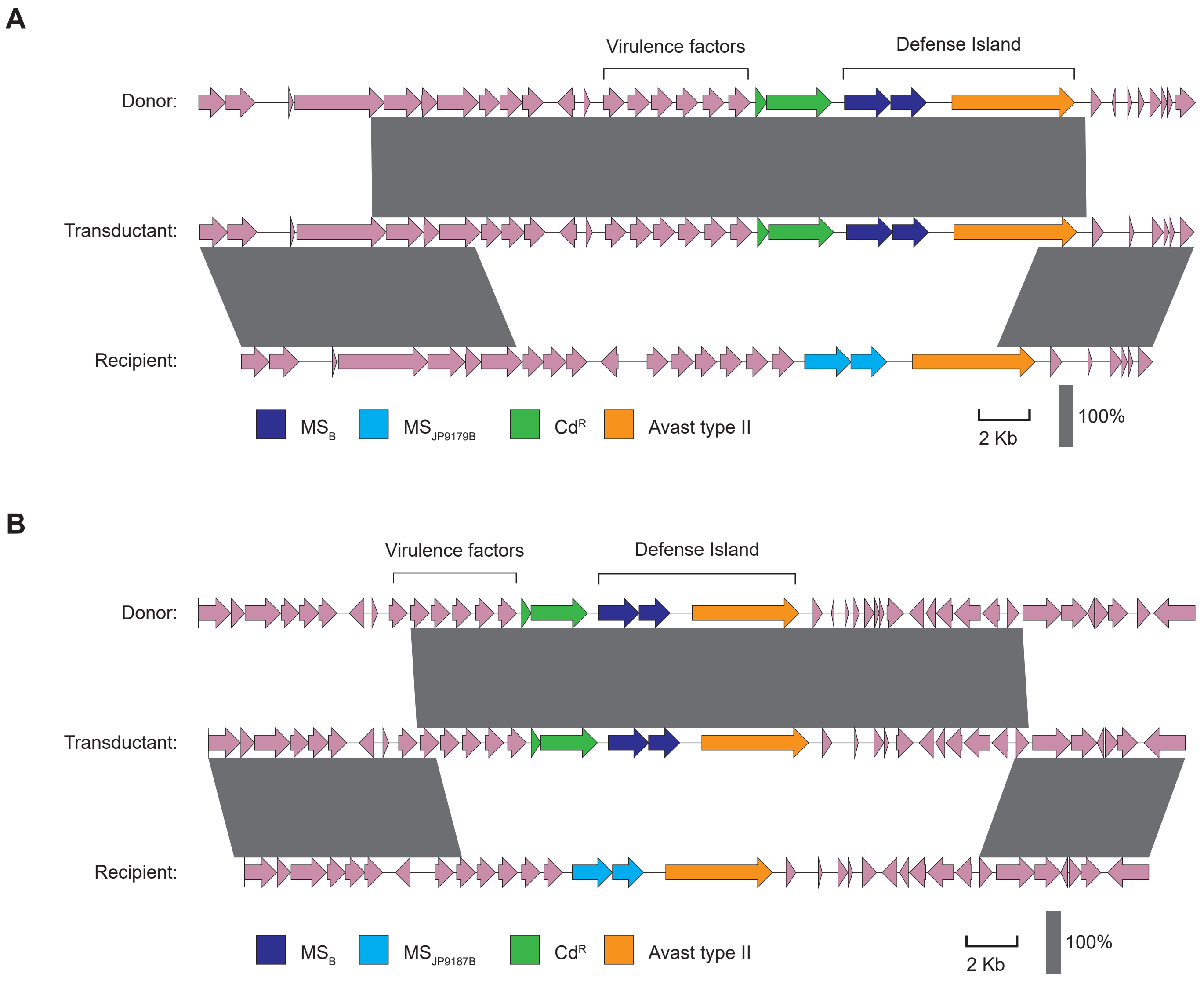

Figure S4. LT of MS$_B$ to different CCs. Genomic organisation of the locus surrounding MS$_B$ (top) from *S. aureus* RN4220 being transferred to bottom recipient strains JP9179 (**A**) or JP9187 (**B**), relating to Fig. 2a. In each case, transductants where the recipient's original MS alleles are replaced with MS$_B$ were generated. The Avast type II system in the defence island was also transferred. The transfer demonstrated the broad mobility of defense islands across recipients from different CCs.

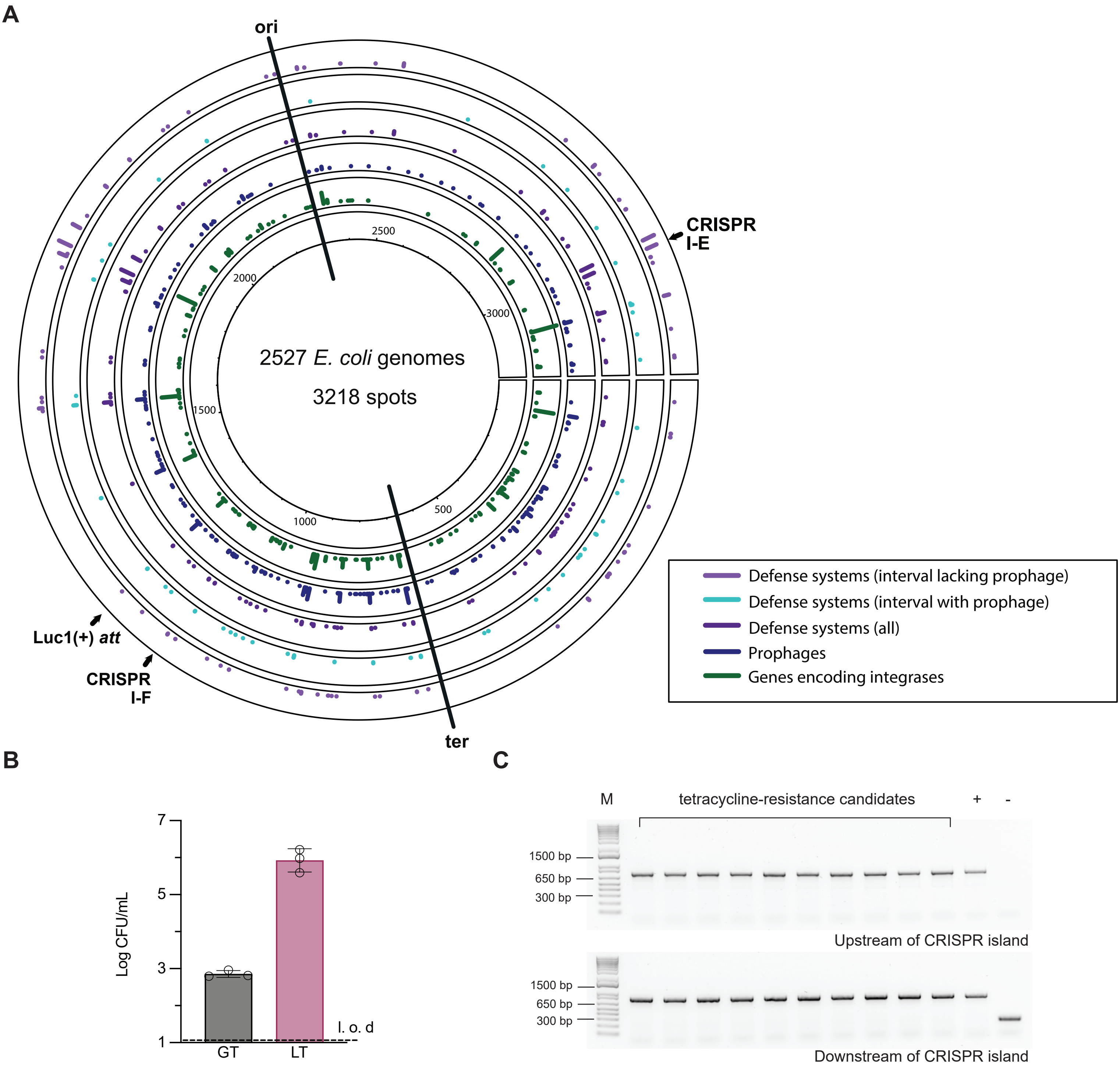

Figure S5. Distribution of LT, defence genes and prophages in *E. coli.* (A) 2,527 complete *E. coli* chromosomes were analysed for the presence of defence systems, prophages and integrases (see methods for details). In total, 3,218 spots where these components are located were identified. From the outer to the inner ring respectively, each circle represents defence systems not in prophages, defence systems located within prophages, all defence systems combined, prophages, and integrase genes. Due to both prophages and defence genes being distributed broadly throughout the genome, and LT being able to mobilise more than 300 kbp per event, practically every defence gene in the genome has the potential to be mobilised by LT. The origin (*ori*) and terminus (*ter*) sequence locations are indicated. **(B)** Phage Luc1(+) efficiently mobilises the CRISPR-Cas island via LT, with GT serving as a control. Transduction efficiencies are represented as log-transformed CFU/mL of donor lysate. Each bar represents the mean of three biological replicates ± SD. l.o.d., limit of detection. **(C)** PCR validation of CRISPR-Cas island transfer, visualised by agarose gel electrophoresis. PCR reactions for the top gel used a primer pair within the CRISPR island which only amplifies after LT. The bottom gel uses a primer pair where one primer is in the chromosome, and one is in the CRISPR island or the empty hotspot. The smaller band in the negative control indicates an empty hotspot. Positive (+) and negative (−) controls were included. M: DNA marker.

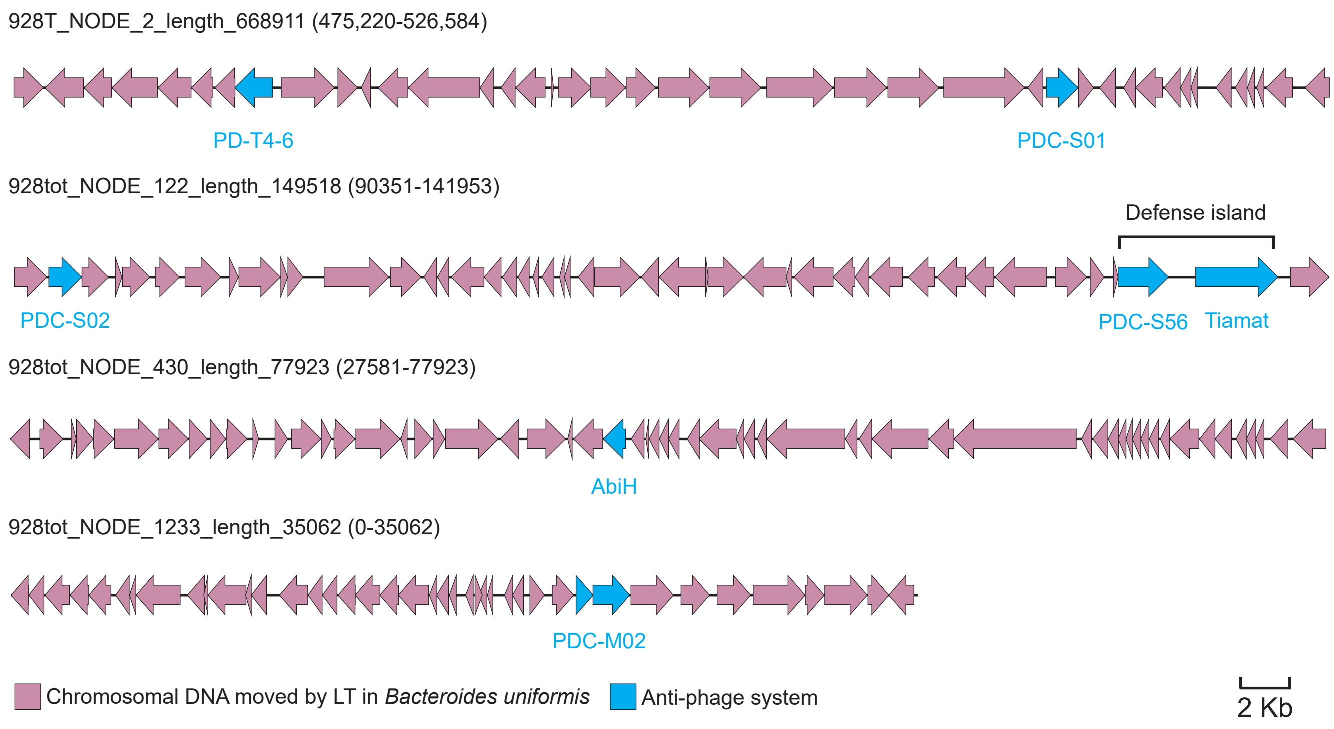

Figure S6. LT of defence genes is observed in human gut. A subset of published metagenomic scaffolds from *Bacteroides uniformis* from faecal samples from human donor 928 showed hallmarks of LT *(49).* DNA was extracted from virus-like particles and sequenced with short-read Illumina sequencing and long-read Oxford nanopore sequencing. For scaffolds that were suggestive of LT (with high coverage of certain chromosomal regions), we analysed the sequences with DefenseFinder *(15)* and PADLOC *(79)* to identify known and putative anti-phage defence systems. This figure shows parts of scaffolds that contained defence genes, highlighted in blue. Identified systems include PD-T4-6 *(6)*, Tiamat *(7)*, and AbiH *(80)*. Predicted but not experimentally confirmed systems include PDC-S01, PDC-S02, PDC-S56, and PDC-M02 *(80)*. Overall, this demonstrates that LT can mobilise defence genes in nature.

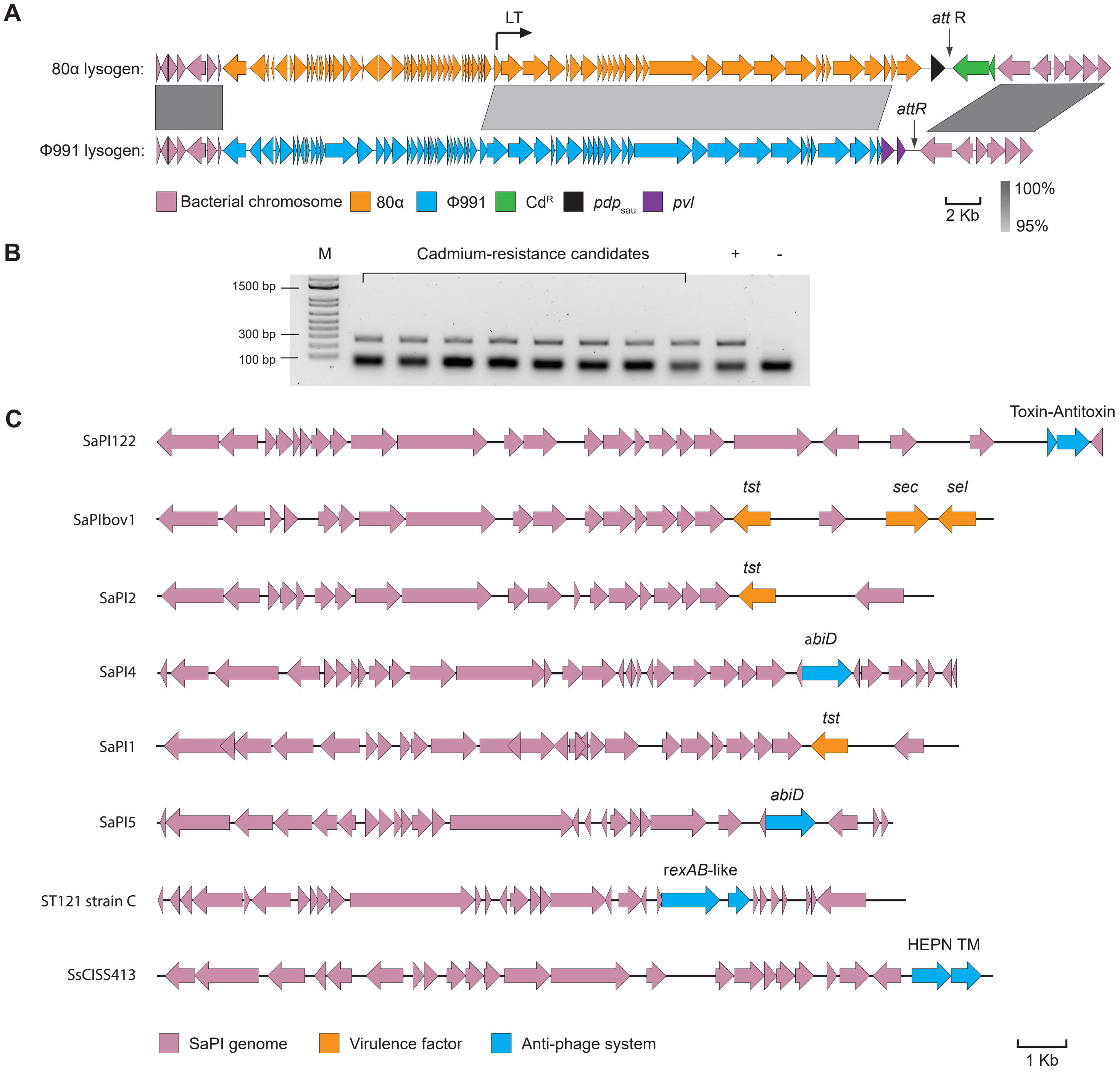

Figure S7. LT can diversify phage and PICI genomes by mobilising the phage/PICI region after *terS* and the adjacent chromosome in the first headful. (A) Schematic representation of the genetic organisation of the 80α and ϕ991 prophages, highlighting the mosaicism and the genetic similarity after their *terS* gene. The genomic region flanking the phage attachment site, at the end of the phage genome (*attR*), shows high similarity, allowing recombination between the incoming LT particle and the recipient lysogenic strain. This allows the exchange of gene pdp_{Sau} from 80α into ϕ991. See Fig. 5a-c for more details. *pvl*, Panton-Valentine leukocidin. **(B)** PCR validation of pdp_{Sau} transfer. Cadmium-resistant transductants from Fig. 5a were screened using PCR and visualised by agarose gel electrophoresis to confirm the presence of pdp_{Sau} in the transductant colonies. The strong lower band is non-specific primer dimers. Positive (+) and negative (−) controls were included. M: DNA marker. **(C)** Genomic organisation of *S. aureus* SaPIs SaPI122, SaPIbov1, SaPI2, SaPI4, SaPI1, SaPI5, and SaPI ST121, and *Staphylococcus saprophyticus* SsCISS413. Virulence factors (orange) and anti-phage systems (blue), which are often encoded near the genome ends, are highlighted. Notable examples include *tst* (toxic shock syndrome toxin) in SaPIbov1 and SaPI2, *abiD* (abortive infection system) in SaPI4 and SaPI1, and the HEPN-TM *(25)* anti-phage system in SsCISS413. Two likely anti-phage systems are Toxin-Antitoxin, containing a predicted Gcn5-Related N-Acetyltransferases (GNAT) toxin and an antitoxin, and *rexAB*-like, which has similarities to the RexAB system. Overall, these genomic features on the right-hand side of the SaPIs suggest a potential role of LT in their acquisition and dissemination, contributing to the dynamic evolution of SaPIs. The scale bar represents 1 kb.

Supplementary Tables

Table S1: Summary of clonal complexes (CCs) used as recipients in inter-CC transfer assays.

Table S2: *Staphylococcus aureus* genomes used in this study.

Table S3: Table showing the number of defence systems from various categories detected in different spotIDs.

Table S4: Defence systems found in the packaged DNA from the gut virome.

Table S5: Phages and phage-inducible chromosomal islands (PICIs) used in this study.

Table S6: Bacterial strains used in this study.

Table S7: Plasmids used in this study.

Table S8: Oligonucleotides used in PCR and cloning experiments.

Table S9: The sites of marker used in *S. aureus* and *E. coli*.
